# Systematic review of prognostic models in Parkinson’s disease

**DOI:** 10.1038/s41531-025-01112-x

**Published:** 2025-08-29

**Authors:** Yan Li, Millie McDonald-Webb, David J. McLernon, Carl E. Counsell, Angus D. Macleod

**Affiliations:** 1 Institute of Applied Health Sciences, University of Aberdeen, Polwarth Building, Foresterhill, Aberdeen, UK; 2 School of Medicine, Medical Sciences and Nutrition, University of Aberdeen, Polwarth Building, Foresterhill, Aberdeen, UK

**Keywords:** Outcomes research, Medical research

## Abstract

Predicting outcomes for people with Parkinson’s (PwP) can enable better information provision, personalised treatments, and enhanced trial design. It is unclear what prognostic models are optimal for use. We systematically reviewed previously published prognostic models for PwP, assessed quality, and made recommendations. We searched MEDLINE and EMBASE for studies developing/validating models predicting clinical outcomes in PwP. We assessed risk of bias and applicability using the PROBAST tool. We screened 1024 references and identified 25 studies (41 prognostic models). The most common outcomes were falls (11 studies), dementia (7) and motor complications (4). Most models made short-term predictions (60% ≤2 years). All studies had concerns about bias, e.g., inadequate population details (*n* = 16), suboptimal methods for missing data (*n* = 21), and no external validation (*n* = 22). 13 models had sufficient information to be used in practice. Further development and validation of prognostic models is needed which follows existing guidelines to reduce risk of bias.

## Introduction

Parkinson’s disease (PD) is a progressive disorder, which often leads to poor outcomes, including falls, dementia, and shortened survival. Being able to predict individualised risk of such outcomes in PD has many advantages: (i) informing people with PD (PwP) how they may be impacted; (ii) improving recruitment, randomisation, and analysis of randomised controlled trials; (iii) enabling clinicians to offer targeted personalised treatment to PwP; and (iv) allowing case-mix correction when comparing outcomes over different hospitals or regions^1,2^. These benefits can best be realised with prognostic models. A prognostic model is a statistical tool which combines an individual’s characteristics to predict the probability of a specific outcome within a period of time.

Given the importance of model validation, it is important to clarify related terminology. Internal validation involves resampling from the same development dataset to test the model performance in the underlying population, while external validation involves assessing model performance in another independent dataset^3^. Calibration and discrimination are measurements of model performance in validation. Calibration refers to the agreement between predicted risks from the model and observed outcomes. Three popular methods to assess calibration are mean calibration (overall observed outcome fraction/average predicted risk), calibration slope (assesses under or over prediction in high/low risk PwP), and calibration plots^4^. Discrimination refers to the model’s ability to distinguish predicted risk between PwP who developed the outcome and those who did not, often measured with the C-statistic^5^.

To date, there has been no published systematic review of prognostic models in PD. A systematic review of studies which identified PD subtypes using cluster analysis has been published^6^, but the aim of these studies is to make group-level, rather than individualised predictions. We therefore performed a systematic review of studies of prognostic models in PD to comprehensively describe existing prognostic models, assess their methodological quality and make recommendations for use in clinical practice.

## Results

**Fig. 1:**
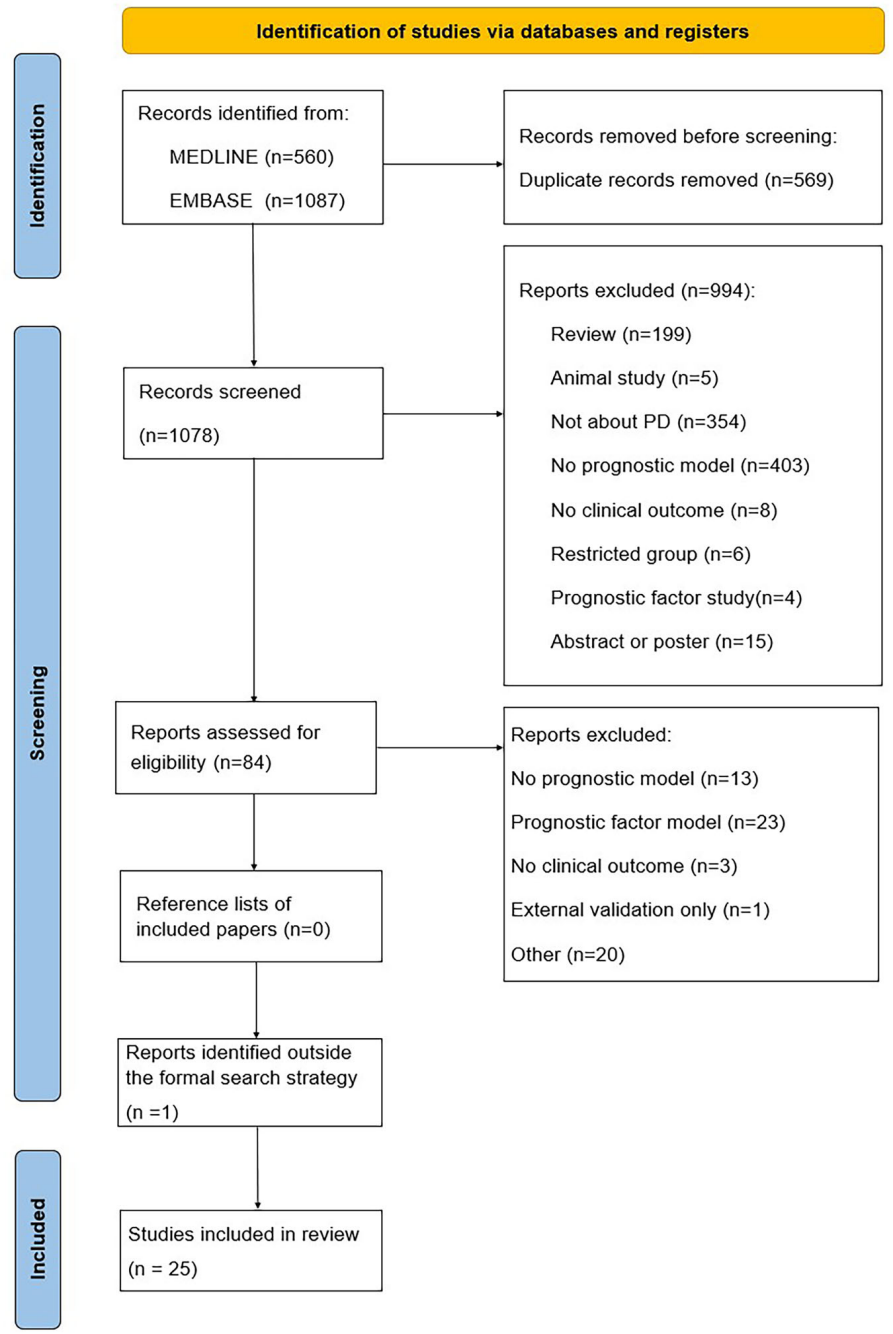
PRISMA flowchart of included studies.

We identified 560 papers in MEDLINE and 1087 papers in EMBASE and one paper was identified outside the formal search strategy. We removed 569 duplicates and excluded 994 papers by abstract and title screening. 84 papers were selected for full text screening. 25 papers^7–31^, comprising 41 prognostic models, were eligible for inclusion (see Fig. 1).

### Study populations and designs

**Fig. 2:**
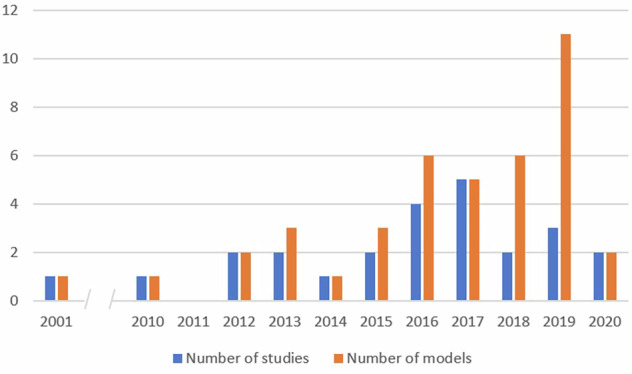
Number of studies and models by years.

15 studies (60%) were published since 2015^7,9,11,14,15,17–20,23,25,27–31^, and one before 2010^8^ (Table 1, Fig. 2). Most studies included European (40%)^8,12,13,15,17,19,20,24,25,28^, North American (12%)^10,23,29^, Australian populations (12%)^11,16,22^, or a combination of these (16%)^14,18,26,27^. 20 studies (80%) were prospective observational cohort studies^7–11,13–17,19–24,27–29,31^ and 7 studies (28%) were inception cohort studies^14,15,19,20,24,27,28^. Models from 7 studies (28%)^14,15,19,20,27–29^ had a defined time-point at which they could be used (i.e. at diagnosis or in early PD) (Table 1). 18 studies (72%)^7–13,16–18,21–26,30,31^ recruited PwP at various disease stages or did not define which PwP were recruited, so we were unable to identify which time-points in the disease course the models were designed to be used. However, one model^23^ recruited PwP with disease durations ranging from 0 to 30 years and included disease duration as a predictor variable in the model, so could potentially be used throughout the disease course if adequately validated.

**Table 1:** Summary of design of studies included in the systematic review of prognostic models in Parkinson’s Disease

Author/ Year	Country	Study type^a^	Type of cohort	Outcome(s)^b^	Sample size	Number of events^b^	Number of predictors^b^	Events per Variable^b^	Disease duration at recruitment in years	Time-point for using the model	Time of outcome or duration of follow-up
Almeida 2016^7^	Brazil	D	Prospective non-inception	M1&2: Recurrent falls	M1&2: 229	M1&2: 84	M1&2: 46	M1&2: 1.8	6.22 (mean)	NS	12 months
Ashburn 2001^8^	UK	D	Prospective non-inception	Falls	63	22	11	2.2	No information	NS	3 months
Custodio 2016^9^	Peru	D	Prospective non-inception	Falls	59	18	15	1.2	6 (median)	NS	1 year
Duncan 2015^10^	US	D	Prospective non-inception	Falls	171	37	14	22	6.6 (mean)	NS	6 months
Ehgoetz Martens 2018^11^	Australia	D	Prospective non-inception	M1: Freezing of gait M2: Freezing of gait	M1: 117 M2: 75	M1: 37 M2: 23	M1: 14 M2: 14	M1: 2.6 M2: 1.6	6.43 (mean)	NS	6-24 months
Exarchos 2012^12^	Greece	D	NS	Multiple symptoms and signs	230	7-128	39	NA	No information	NS	No information
Gervasoni 2015^13^	Italy	D	Prospective non-inception	M1: Falls M2: Recurrent falls	53	M1: 32 M2: 22	M1: 17 M2: 17	M1: 1.9 M2: 1.3	6 (median)	NS	6 months
Gu 2020^14^	US and Europe	D	Prospective inception	Depression	312	36	23	1.6	0.4 (median)	Newly diagnosed PD	2 years
Kelly 2019^15^	UK	V	Prospective inception	Dyskinesia	62	30	5	6	1.7 (mean)	Newly diagnosed PD	200 weeks
Kerr 2010^16^	Australia	D	Prospective non-inception	Falls	130	48	49	1	6.1 (mean)	NS	6 months
Lindholm 2016^17^	Sweden	D	Prospective non-inception	M1&2: Falls	M1&2: 135	45	3	M1&2: 15	4 (mean)	NS	6 months
Liu 2017^18^	N America & Europe	D + V	Mixed	Global cognitive impairment and dementia within 10 years from onset	D: 1350 V: 1132	D: 168 V: 146	D: 9 V: 9	D: 18.7; V:16.2	NS	NS	10 years
Lo 2019^19^	UK	D	Prospective early disease	Falls, freezing of gait, postural instability, difficulty doing hobbies, cognitive impairment, dependency	237	11-41	998	approx. 0	3.5 (mean)	Early PD	18 months
Macleod 2018^20^	UK (D) Norway (V)	D + V	Prospective inception	M1&2: Mortality M3: Dependency M4: Death/dependency	M1&2: D: 198; V: 192. M3: D:176; V:162. M4: D: 176; V: 162	M1&2: D: 97; V: 37 M3: D: 130; V: 78 M4: D: 154; V: 88	M1&2: D: 8; V: 8 M3: D: 8; V: 8 M4: D: 8 V: 8	M1&2: D: 12.1; V: 4.6 M3: D: 16.3; V: 9.8 M4: D: 19; V: 11	D: 1.1 (median) V: 1.7 (median)	Newly diagnosed PD	D: Up to 12 years; V: up to 8 years
Mak 2014^21^	Hong Kong	D	Prospective non-inception	Recurrent falls	144	42	13	3.2	7.8 (mean)	NS	12 months
Paul 2013^22^	Australia	D	Prospective non-inception	Falls	205	125	25	5	7.3 (mean)	NS	6 months
Phongpreecha 2020^23^	US	D	Prospective non-inception	Normal cognition; MCI; Dementia	827	Normal cognition: 208 MCI:160 Dementia:459	22	Normal cognition (9.5); MCI (20.9); Dementia (7.3)	9 (mean)	Whole disease course	1–2 years
Pouwels 2013^24^	UK	D	Prospective inception	M1: Osteoporosis M2: Hip fracture risks	1&2: 4411	NS	26	NA	No information	NS	Average 4 years
Redensek 2019^25^	Slovenia	D	Retrospective	M1&2: Motor fluctuations M3&4: Dyskinesia	M1&2: 231 M3&4: 231	M1&2: 120 M3&4: 96	M1&2: 50 M3&4: 50	M1&2: 2.4 M3&4: 1.92	No information	NS	Median Follow-up time: 7.1 years
Schapira 2012^26^	Multiple	D	RCT	Dyskinesias (NA)	NS	NS	5	NA	2 (mean)	NS	134–208 weeks
Schrag 2017^27^	Mulitple	D	Prospective inception	Cognitive impairment	390	52	22	2.4	0.33 months (mean)	Newly diagnosed PD	2 years
Velseboer 2016^28^	D: Netherlands; V: UK	D + V	Prospective inception	Composite outcome (instability, dementia, or death)	D: 111; V: 108	D: 54; V: 65	D: 14; V: 14	D: 3.9; V: 4.6	D: 0.3 (mean) V: 0.3 (mean)	Newly diagnosed PD	5 years
Wang 2017^29^	US	D	RCT	Need for levodopa treatment	755	NS	5	NA	1.1 (mean)	Untreated early PD	24 months
Wang 2017^30^	NS	D	Retrospective	Imbalance	76	12	8	1.5	5.1 (mean)	NS	36 months
Ye 2017^31^	South Korea	D	Prospective non-inception	Dementia	216	52	26	2	3.3 (mean)	NS	Mean Follow-up time:2.7 years

*dev* development, *DRT* Dopamine replacement therapy, *H&Y* Hoehn and Yahr, *M* model number, *MCI* mild cognitive impairment, *MEAMS* Middlesex Elderly Assessment of Mental State, *MSE* Mini-Mental State Examination, *NA* not applicable, *NS* not stated, *RCT* randomised control trial, *val* validation.

^a^Types of prediction modelling studies based on CHARMS checklist (cite): D=prediction model development without external validation in independent data; D + V=prediction model development with external validation in independent data, V external model validation (may with model updating).

^b^Initial numbers, where present, designate multiple models in one paper.

### Outcomes of study

The most common prognostic outcome was falls/recurrent falls, which was predicted in 11 studies (44%)^7–10,12,13,16,17,19,21,22^. 7 studies (28%)^12,18,19,23,27,28,31^ predicted cognitive impairment/dementia, 4 studies (16%)^12,15,25,26^ predicted motor complications, 3 studies (12%)^11,12,19^ predicted freezing of gait, 3 studies (12%) predicted imbalance^12,19,30^, 2 studies (8%)^18,20^ predicted functional disability, 2 studies (8%)^20,28^ predicted a composite poor outcome, and single studies predicted depression^14^, mortality^20^, fracture risk^24^, difficulty doing hobbies^19^, and several other symptoms and signs^12,29^. The follow-up duration over which predictions were made varied from 3 months^8^ to 12 years^20^, most of which were <2 years (60% of models) and 4 studies^18,20,25,28^ had 5 or more years’ follow-up (Table 1).

### Predictors in study

**Fig. 3:**
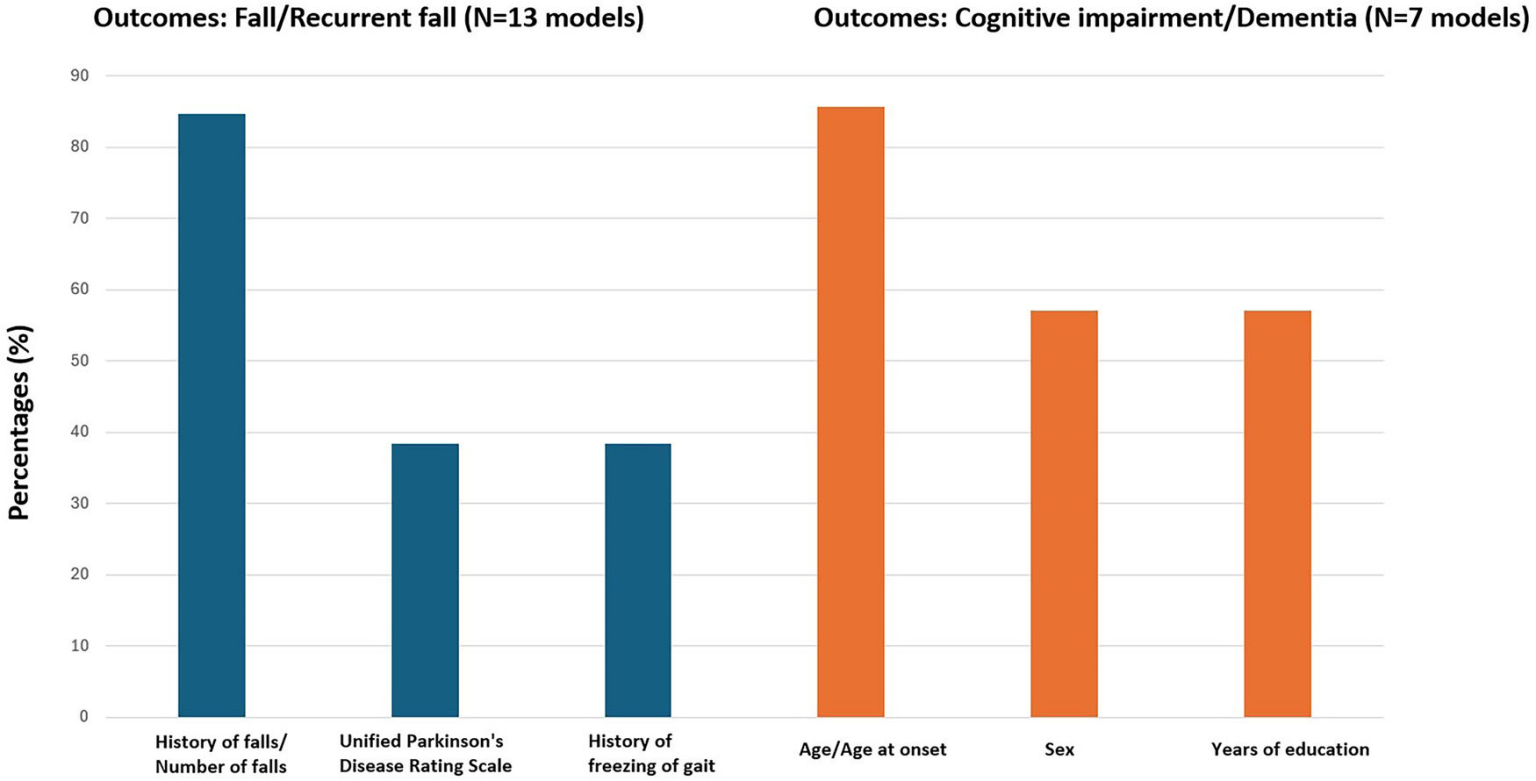
Proportion of models including the most commonly used predictors (data shown for the two most frequent model outcomes; variables appearing in less than a third of the models are not shown).

The number of predictors per model ranged from 3 to 998 (Table 1). 17 studies comprising 24 prognostic models (59%) used variables which were simple to collect in clinical practice, but 7 studies comprising 11 prognostic models (27%) included predictors that are not always routinely available in clinical practice, such as DAT imaging measurements, CSF biomarkers, or genetic polymorphism data (supplementary Table 1)^13,14,18,23,25,27,31^. In one study, 6 models (15%) were based on smartphone features and the corresponding app/analysis pipelines are not available for routine use in clinical practice^19^. 8 studies dichotomised or categorised continuous/discrete predictors^7,10,12,13,17,22,24,31^. Across 24 studies with 35 final models which specified the predictors, the most common predictors were age/age at onset (*n* = 25), sex (*n* = 15), and original or Movement Disorder Society Revision of the UPDRS (*n* = 12) (supplementary Table 2). In Fig. 3 we showed the percentages of predictors included in the models for the two most common outcomes (falls/recurrent falls [13 models] and cognitive impairment/dementia [7 models]). We question the usefulness of previous falls as a predictor of future falls, as was the case in 11 models^7–10,13,17,21,22^ because once PwP have started to fall, the fracture risk is already present and physiotherapy interventions for falls and balance are already indicated.

### Study sample sizes

5 studies (20%) had fewer than 100 participants^8,9,13,15,30^ (Table 1). Only 4 studies (16%) had an events per variable (EPV) of at least 10^10,17,18,20^ (Table 1), the usual rule of thumb for minimum EPV required for Cox or logistic regression modelling^32^, and many of the other studies had EPVs much less than 10^7–9,11,13,14,16,19,25,27,28,30,31^. 4 studies (16%) didn’t give information about the number of events^18,24,26,29^ (Table 1).

### Model development

12 studies (48%) did not provide information on the number of participants lost to follow-up^9–12,15,18,20,22,24–26,29,31^ and 11 studies (44%) didn’t report the number of participants with missing data^9,11,12,15–17,21,22,24,26,31^ (supplementary Tables 3 and 4). 10 studies (40%) gave full information of missing data (number and imputation method)^7,10,13,14,18,23,25,27–29^. The most common method of handling missing data was complete case analysis (28%)^7,10,13,15,18,25,29^. 2 studies (8%) handled the missing data with multiple imputation^14,28^ (Table 2). 8 studies (32%) transformed continuous predictors into dichotomous or category variables^7,10,12,13,17,22,24,31^ and 10 studies (40%) selected predictors by univariable analysis^7,9,13,14,16,20,22,25,27,31^ (supplementary table 1 and 5).

**Table 2:** Summary of modelling methods and validation

Author/year	Methods to handle missing data	Modelling method	Calibration; discrimination methods	Model performance	Model presentation (sufficient/insufficient for model use in practice)
Almeida 2016^7^	Complete case	Cox regression	Not done; C-statistic	Good discrimination (AUC around 0.8)	Only coefficients (insufficient)
Ashburn 2001^8^	Not stated	Logistic regression	Not done; Not done	Not reported	Only coefficients (insufficient)
Custodio 2016^9^	Not stated	Logistic regression	H-L test; C-statistic	Limited statistical power on calibration; good discrimination (AUC = 0.93)	Only coefficients (insufficient)
Duncan 2015^10^	Complete case	Logistic regression	H-L test & calibration table; C-statistic	Limited statistical power on calibration; good discrimination (AUC = 0.83)	Only coefficients (insufficient)
Ehgoetz Martens 2018^11^	Not stated	Logistic regression	H-L test; Not done	Limited statistical power on calibration	Full equation (sufficient)
Exarchos 2012^12^	Not stated	Decision tree	Not done; not done	Not reported	No information (insufficient)
Gervasoni 2015^13^	Complete case	Logistic regression	Not done; C-statistic	Fair to good discrimination (AUC = 0.72–0.84)	Only coefficients (insufficient)
Gu 2020^14^	Multiple imputation	XGBoost & logistic regression	Calibration plot & H-L test; C-statistic	Good calibration in logistic regression and underpredicted in XGBoost; good discrimination both models (AUC > 0.9)	Full equation (sufficient)
Kelly 2019^15^	Complete case	Cox regression	Not done; C-statistic	Fair discrimination (AUC = 0.68)	Not applicable (external validation study)
Kerr 2010^16^	Not stated	Logistic regression	Not done; C-statistic	Fair discrimination (AUC = 0.74)	No information (insufficient)
Lindholm 2016^17^	Not stated	Logistic regression	H-L test; not done	Limited statistical power for calibration; not reported.	Only coefficients (insufficient)
Liu 2017^18^	Complete case	Frailty Cox model	Not done; C-statistic	Good discrimination in global cognitive impairment and dementia (AUC > 0.8)	Online risk calculator available (sufficient)
Lo 2019^19^	Not stated	Random forest	Not done; C-statistic	Good discrimination in all 6 outcomes (AUC around 0.8)	No information (insufficient)
Macleod 2018^20^	Single imputation	Weibull model	Calibration plot; C-statistic	Good calibration, fair discrimination (AUCs around 0.75)	Full equation (sufficient)
Mak 2014^21^	Not stated	Logistic regression	Not done; Not done	Not reported	Subset equation of full model (insufficient)
Paul 2013^22^	Single imputation	Logistic regression	H-L test & calibration table; C-statistic	Limited power for calibration; good discrimination (AUC around 0.8)	Only coefficients (insufficient)
Phongpreecha 2020^23^	Restricted Boltzmann machine	Generalised multitask	Not done; C-statistic	Unclear discrimination performance (range of C-statistics only)	No information (insufficient)
Pouwels 2013^24^	Not stated	Cox regression	Not done; C-statistic	Fair discrimination (AUC around 0.7)	No information (insufficient)
Redensek 2019^25^	Complete case	Cox regression	Not done; C-statistic	Fair discrimination (AUC around 0.7)	Only coefficients (insufficient)
Schapira 2012^26^	Not stated	Cox regression	Not done; C-statistic	Fair discrimination	Full equation and online risk calculator (sufficient)
Schrag 2017^27^	Single imputation	Logistic regression	H-L test; C-statistic	Limited power for calibration; good discrimination (AUC around 0.8)	Full equation (sufficient)
Velseboer 2016^28^	Multiple imputation	Logistic regression	Calibration plot & slope and H-L test; C-statistic	Good calibration; fair discrimination in internal validation (AUC = 0.75) and good discrimination in external validation (AUC = 0.85)	Full equation (sufficient)
Wang 2017^29^	Complete case	Joint modelling	Not done; C-statistic	Fair discrimination (AUC = 0.75–0.79)	Full equation (sufficient)
Wang 2017^30^	Not stated	Bayesian linear mixed-effects model	Not done; C-statistic	Good discrimination (AUC = 0.99)	Full equation (sufficient)
Ye 2017^31^	Not stated	Cox regression	Not done; C-statistic	Good discrimination (IAUC = 0.79)	Only coefficients (insufficient)

*AUC* area under the receiver-operator curve, *H-L* Hosmer-Lemeshow, *IAUC* incremental Area Under Curve.

12 studies (48%) used logistic regression^8–11,13,14,16,17,21,22,27,28^ and 3 studies (12%) used machine learning (decision trees, XGBoost, and random forests) to build the prognostic model^12,14,19^. None of the machine learning models reported key predictor importance (e.g., SHAP values) or provided sufficient details for independent validation.8 studies (32%) didn’t account for censoring and simply excluded censored participants in the analysis^8,13,14,16,17,21,27,28^. 10 studies (40%) used time-to-event survival analysis to build the prognostic models: 6 studies used Cox regression^7,15,24–26,31^. Other studies used a frailty Cox model^18,23^, Weibull parametric survival model^20^ and a dynamic prediction model^29^ (Table 2). Three studies reported checking the proportional hazards assumption in survival analysis^7,18,20^ (Table 2 and supplementary table 5).

### Model evaluation and performance

Two studies^10,17^ that aimed to externally validate previously published models did not use the original model equation to make predictions for PwP in their validation dataset^3^. Therefore, these 2 studies^10,17^ were not truly external validation studies. We classed these studies as model development in the PROBAST assessment (Tables 1 and 3).

**Table 3:**
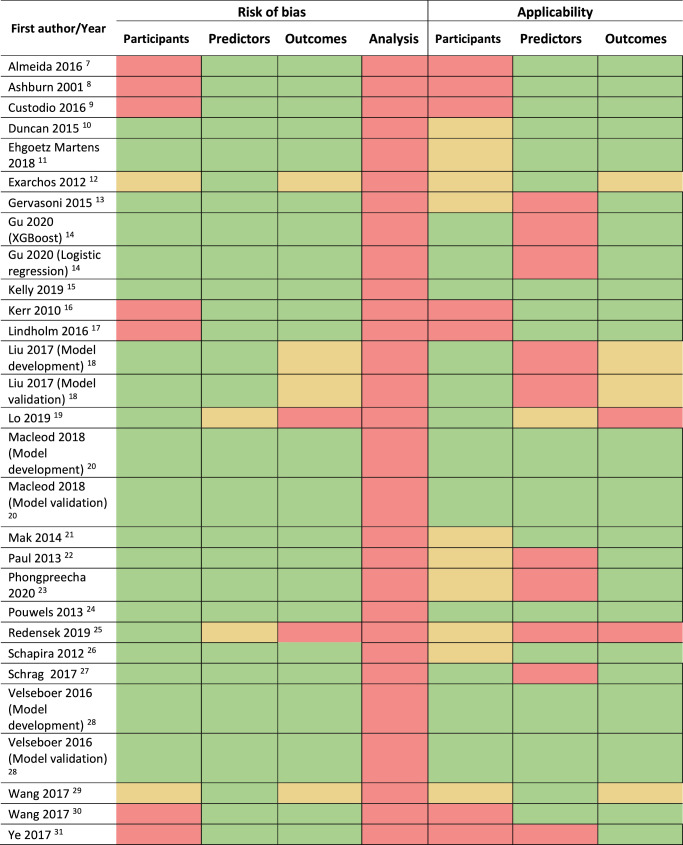
Summary of risk of bias and applicability in PROBAST

Guide to colour shading in the following PROBAST tables.

Internal validation and model equation assessment only applies to model development studies (*n* = 24) (Table 1). 7 studies (28%) didn’t perform internal validation^8–11,17,21,26^, 7 studies (28%) didn’t provide clear information about whether internal validation had been applied in all model development procedures or not^13,14,16,23,24,29,31^, and 3 studies (12%) used split data methods^14,27,30^ (supplementary Table 6). 15 studies (60%) used cross-validation or bootstrap resampling to assess optimism in model performance^7,12,13,16,18–20,22–25,27–29,31^ (supplementary Table 6). Only 3 studies (12%) performed both internal and external validation after model development^18,20,28^ (supplementary Table 6). One study^18^ didn’t give the number of events in the development and validation datasets (Table 1).

3 studies (12%) didn’t evaluate model performance^8,12,21^ (supplementary Table 7). 12 studies (48%) reported internal discrimination performance but did not report calibration performance^7,13,16,18,19,23–26,29–31^ and one external validation study^15^ reported the discrimination performance without reporting calibration (Table 2). 6 studies (24%) used the Hosmer-Lemeshow goodness-fit-test to assess the internal calibration performance^9–11,17,22,27^ (supplementary Table 7). One study (4%) used both calibration plot and slope to present models’ internal and external calibration performance^28^, one study (4%) used calibration plot to present models’ internal and external calibration performance^20^ and one study (4%) used calibration plot to present models’ internal calibration performance^14^ (supplementary Table 7).

### Model reporting

9 studies (36%) including 13 models (32%) gave sufficient information for the models to be used in clinical practice^11,14,18,20,26–30^ (Table 2). 10 studies (40%) did not report the intercept or baseline hazard^7–10,13,17,18,22,25,31^. 5 studies (20%) did not provide the model equation or sufficient details to replicate the model^12,19,21,23,24^ and one study provided a plot of estimated coefficients instead of giving specific values^16^.

### Risk of bias/applicability

We found 8 studies (32%) which had inclusion and exclusion criteria that would be broadly generalisable to unselected populations with PD^14,15,18–20,24,27,28^ (supplementary Table 8), which had low concern of applicability (supplementary Table 9). 16 studies (64%) lacked details of important aspects of study design (e.g. recruitment methods/dates, diagnostic criteria)^7,8,10–13,16,17,21–23,25,26,29–31^ and 7 studies (28%) had selection concerns that could bias the studies towards healthier participants (e.g., excluding on the basis of comorbidities, older age) raising concerns about generalisability or risk of bias^7–9,16,17,30,31^ (supplementary Table 8, 9 and 10).

Supplementary Table 11 contains the risk of bias results relating to the predictors studied. One study (4%) had risk of bias in the predictors as they used a retrospective cohort without stating how subjective predictors (e.g., depression, olfactory dysfunction) were measured^25^. 7 studies (28%) included predictors that may not be routinely available in clinical practice, such as CSF biomarkers or imaging data^13,14,18,23,25,27,31^ so these models may not be feasible in clinical practice, especially in resource-poor settings.

For the risk of bias relating to the outcomes in studies, one study (4%) had unclear risk of bias as it didn’t state the outcome definition^12^ (supplementary Tables 12 and 13). Outcome definitions in 2 studies (8%) may have been biased by determination with knowledge of predictor information as the outcome definitions were subjective^19,25^ (supplementary Tables 12 and 13).

## Discussion

We identified 25 prognostic model studies, comprising 41 prognostic models, which have been published with the aim of predicting the individualised risk of future outcomes in PD. A wide range of clinical outcomes were used in these studies and the most common outcome was falls/recurrent falls. Most models made short-term predictions. None of the prognostic models had low risk of bias. The common analysis issues leading to risk of bias were potential mishandling of missing data including incorrect missing data imputation (potentially leading to biased predictions and biased model performance); selecting predictors using univariable screening and risk of overfitting from low EPV ratios (leading to both biased model performance from over-estimated discrimination performance and also biased predictions due to overestimation in those at higher risk of the outcome and underestimation in those at lower risk of the outcome^33^); and the lack of external validation (leading to potential bias in model performance if used in different populations). Many of the included studies did not provide sufficient details of the models to enable use in clinical practice or research.

The review showed that some studies omitted to give basic information about the study population, which made it difficult to assess selection bias and applicability. Other studies had selection biases which led to study populations being skewed towards healthier subjects. Most studies were performed in Europe, the United States and Australia, so non-Caucasian populations are under-represented.

Half of the studies didn’t report the number of participants lost to follow-up. As PD is slowly progressive, there will often be losses to follow-up with long follow-up durations. Most models had too many predictors for the number of events, which carries a high risk of overfitting^32,34^, and therefore high risk of poor performance.

The recommended method for handling missing data when data are missing at random is multiple imputation^32^. Missing at random means that systematic differences between the observed and missing data can be explained by associations with the observed data^35^. In this scenario, using single imputation or deleting participants with missing values and conducting a complete cases analysis may cause a selection bias. 12 studies did not mention anything about missing data^8,9,11,12,16,17,19,21,24,26,30,31^, 8 studies deleted observations with missing data or used single imputation with no justification^10,13,15,18,20,22,25,27^, 2 studies assume missing at random but deleted missing data^7,29^, 1 study imputed missing data with Restricted Boltzmann machine with adequate justification^23^, and 2 studies used multiple imputation with no justification^14,28^. Researchers should be aware that multiple imputation may lead to biased results when data is not missing at random and that a complete case analysis may be appropriate even when data are not missing completely at random^36^.

Time to event models assume that censoring is uninformative, i.e., that the probability of being censored is independent of the outcome (i.e. the probability of getting the outcome in those who are censored is the same as those who remain under follow-up). An example of the probability of being censored being related to the outcome is patients who drop out having more severe disease than those who remain under follow-up. The missing survival times would likely be systematically shorter than survival times in those who remain, resulting in biased estimates. In our review we only found one paper^7^ that reported the number of patients lost to follow-up. There were only 4 patients lost to follow-up and the reason for the loss was not stated. While it is not clear whether this censoring was uninformative, the small number lost means it is unlikely to bias the predictions. All other studies that used time-to-event methods to account for censoring did not provide information about censored patients. We suggest that researchers report the number of patients censored before the end of study (i.e. non-administrative censoring) and if possible, provide reasons why. Methods to account for informative censoring include using inverse probability weights in the Cox model or joint models, which should be considered in studies with higher rates of loss to follow-up^37–39^.

None of the studies considered competing risks in their analysis. Competing risks occurs when one or more events precludes the occurrence of the event of interest. Ignoring them can result in biased predictions. Competing risks can be accounted for using methods such as stratified Cox regression or the Fine and Grey model^40^.

Several studies dichotomised or categorised continuous variables which may lose information and reduce predictive performance^32,41^. Most studies selected predictors inappropriately with univariable analysis or backward/forward selection. A predictor which has no association with the outcome in univariate analysis, may become statistically significant in the multivariable analysis due to confounding^42,43^. It is recommended that known clinically important predictors should be included in the modelling regardless of statistical significance^32^. This is because selection of predictors based on statistical significance such as backward/forward selection methods can lead to model overfitting, miscalibrated risks, and biased predictions^33^. In our view, the selection of predictors should primarily be based on clinical knowledge rather than solely on statistical significance. We recommend that researchers collaborate with clinicians to select predictors, combining clinical and statistical expertise. If it is known from previous research or clinical knowledge that a predictor is associated with the outcome, even if not statistically significant, it should still be included in the analysis^42^.

The performance of most prognostic models was unclear and many lacked external validation, which is essential before a model can be applied in clinical practice^44^. Half of the studies only reported discrimination performance by C-statistic which provides limited information (a high C-statistic may still lead to poor estimation of absolute risk^45^). Ideally a prognostic model would predict an individual’s risk of a specific outcome within a period of time. However, two papers^7,24^ stratified patients into different risk groups rather than estimate individual predictions. In such cases, reporting only the C-statistic may be sufficient. However, for the other 10 studies^13,16,18,19,23,25,26,29–31^ which did develop a model to provide individual predictions then the C-statistic is not enough to assess predictive performance. Without also assessing calibration performance, we cannot determine how well the predicted probabilities align with observed outcomes. Calibration performance is critical for ensuring that a prognostic model’s predictions are accurate and reliable, which is essential for clinical decision-making. For calibration performance, most studies only used the Hosmer-Lemeshow test which has limited statistical power to evaluate miscalibration^32^. 3 studies used calibration plots or slope to present their model’s calibration performance as recommended and no study used the gold standard approach (flexible calibration plot) to assess calibration^46^.

While prognostic factor studies only need to report the estimated coefficients of predictors, a prognostic model study must report additional details (e.g. the constant) so that the model can be replicated by independent researchers to perform external validation or for clinicians to predict probabilities in clinical practice. 8 studies’ models gave full model details although another study’s model presented an online risk calculator, which could be applied in clinical practice.

None of the included studies had low risk of bias, as per the PROBAST criteria, so we cannot recommend any models without reservations. It is vital that models are externally validated to demonstrate generalisability before use in contexts other than the local geographical context in which it was originally developed^43^. Only 3 studies with external validation reported sufficient information for the models to be used by other researchers or clinicians, and therefore could be considered for use in practice, ideally following further validation work^18,20,28^. These models all had some concerns about bias relating only to the analysis domain (potentially leading to bias in predictions and in model performance).

The first of these is the prognostic model predicting risk of dementia by ten years by Liu et al.^18^ who performed individual-participant-data meta-analysis of nine prospective cohorts with a very large sample size using a frailty Cox model to account the heterogeneity between studies^47^. The study didn’t report calibration performance and used complete case analysis for missing data. We recommend that calibration is fully assessed in future validation studies for this model. Another issue about the model’s use in clinical practice is the fact that predictor information was collected at widely varying disease durations, without a variable for disease duration in the model, so it is unclear when it is valid to use this model, although the majority (61%) of participants were recruited within two years from diagnosis. Although measures of disease severity may account, to a degree, for differences in disease duration, rates of disease progression over time vary substantially between individuals. Therefore, a combination of a disease duration variable together with disease severity is important in a prediction model^2^. Further work to clarify the validity in inception cohorts is needed.

The second is a set of prognostic models predicting functional dependency, mortality, or a composite outcome “death or dependency” by Macleod et al.^20^. This study developed parametric survival models in a UK incidence cohort and performed external validation in a Norwegian incidence cohort. This model had reasonable discrimination and showed a calibration plot with lower baseline risk in the Norwegian cohort. The authors reported recalibrated values of the model which could be used in the Norwegian setting. Concern about risk of bias relate to the use of univariable analysis for predictor selection, and low events per variable ratios in the validation cohort. Further validation of this model in a larger cohort would therefore be useful.

The third is a prognostic model to predict a composite poor outcome at five years from diagnosis by Velseboer et al.^28^, developed in an inception cohort in the Netherlands using logistic regression, with external validation in a UK incidence cohort. The model demonstrated good discrimination (C-statistic 0.85) and adequate calibration (calibration slope 1.13) in external validation. There were some concerns about risk of bias due to their use of logistic regression, which does not account for censoring, and the low events-per-variable ratio raising concerns about overfitting. Further validation in larger cohorts would again be useful.

These models may be of use in research, for example in stratification in clinical trial randomisation, for adjustment for confounding in analysis of randomised controlled trials, or for case-mix correction. However, the use of prognostic models in clinical practice can potentially lead to harms as well as benefits so we hesitate to recommend their use for individual prognostication for PwP, given their limitations, without further external validation followed by rigorous testing to ensure any benefits of using model predictions in clinical care are not outweighed by harms.

This is the first systematic review of prognostic models in PD that aimed to make individual-level predictions. The main strength of this review is that we assessed studies’ quality rigorously using the PROBAST checklist. Other strengths include identifying studies with all types of clinical outcomes, not using language restriction, and using a comprehensive search strategy in multiple databases, displaying the results of the screening process using a PRISMA flow diagram.

There are also limitations of this review. The main limitations are lack of searching of grey literature and not contacting other authors for missing information in the included reports. Due to the time taken to perform this review and prepare it for publication, the searches are now over three years out of date. An updated search carried out on 05/02/2025 found 1118 additional papers in MEDLINE and EMBASE, representing a 104% increase, so future work is needed to update this review.

None of the prognostic models we identified had low risk of bias for all aspects of the study design so there is clearly a need for further prognostic modelling studies in PD. There is clear guidance for carrying out prognostic models, including a reporting checklist (TRIPOD)^48^ and practical guidance for assessment of prognostic model performance and clinical usefulness^4^, and these should be considered in the design, analysis, and reporting stages of future prognostic modelling studies. We would draw attention to recent research regarding sample size calculations for prognostic modelling studies^49^.

To enable prognostic models to be used in clinical settings, regardless of the prediction performance, we recommend researchers give full details about their data source (recruitment methods and dates, diagnostic and inclusion/exclusion criteria) and clear definitions of outcomes and predictors. We strongly recommend researchers present the full equations of prognostic models so they can be replicated or used by others. It is also important that researchers reporting prognostic model development make clear what time point in the disease course the models are to be used (e.g. at diagnosis or at another specified time point). Furthermore, to enhance the feasibility of clinical use of prognostic models, we recommend researchers choose predictors that are routinely available in clinical practice, unless there is clear additional prognostic value of particular biomarkers that are more expensive or invasive to collect. When models are used in clinical practice it is important to evaluate the impact of the model. We did not find any papers describing the use of prognostic models in clinical practice or evaluating the impact of any prognostic model in PD.

In conclusion, there are many methodological shortcomings in existing prognostic model studies in PD and many were published with insufficient detail to allow them to be used by other researchers or clinicians. We have made recommendations for the limited use of three prognostic models that have been externally validated but these all have some concerns about risk of bias and are probably not appropriate for individual use at present without further evaluation. There is therefore a pressing need for further prognostic model development and validation studies using high quality methodology to ensure low risk of bias and for clinical use of high-quality models to be evaluated thoroughly before widespread use.

## Methods

### Literature search

We searched MEDLINE (1946 to latest update) and EMBASE (1947 to latest update) on 20 Feb 2021 to identify primary articles that developed and/or validated prognostic models in PD. The search strategy is detailed in Supplementary Appendix 1.

### Eligibility criteria

We sought to include all published studies of prognostic models in PD predicting clinical outcomes. We did not set inclusion/exclusion criteria relating to timing or definition of outcomes other than to exclude models predicting surrogate measures of outcomes such as measurement scales (e.g. impairment or cognitive scales) or imaging changes. No language restriction was applied.

PD subtyping studies which did not aim to make individualised predictions were excluded. We also excluded prognostic models for use in highly selected groups of PwP, such as those with deep brain stimulation.

### Screening process

References were imported into Endnote and de-duplicated. Two reviewers independently reviewed titles and abstracts for eligibility (YL, MM). The full text papers of the articles were obtained for relevant studies or where relevance was unclear from the abstract. Full text papers were assessed by the same two reviewers independently. Disagreements on inclusion/exclusion of full text papers were discussed with a third or fourth reviewer (ADM, DJM). Reference lists of included papers were reviewed to identify any relevant papers missed from the database searches.

### Data extraction

Two reviewers independently performed the data extraction and recorded it in an electronic data collection form using Microsoft Excel (YL and either MM, ADM, or DJM). Any disagreement was discussed with another reviewer (ADM or DJM). The data extraction form was based on CHARMS (CHecklist for critical Appraisal and data extraction for systematic Reviews of prediction Modelling Studies) checklist^50^ and risk of bias assessment using PROBAST (Prediction model Risk Of Bias ASsessment Tool) checklist^32^. We categorised models into three groups (model development only; model development with external validation; and external validation with or without model updating) and extracted 10 domains based on the CHARMS list from each model:

1. Study location and data source;
2. Recruitment methods, diagnostic criteria;
3. Inclusion/exclusion criteria;
4. Outcomes predicted, follow-up duration, losses to follow-up;
5. Prognostic factors;
6. Sample size, events per variable;
7. Missing data frequency and methods for dealing with missing data.
8. Model development methods;
9. Model performance: internal validation methods, results of calibration, and discrimination.
10. Model evaluation: whether external validation was done and results of external calibration and discrimination.

### Synthesis

We assessed the risk of bias and applicability of published prognostic models using the PROBAST tool and tabulated key aspects of study design, model development, model validation, and risk of bias. We tabulated the most commonly used prognostic factors from the studies. We made recommendations about the usefulness of existing prognostic models. Lastly, we made recommendations for future prognostic model development.

### Registration

The protocol of this systematic review is registered in PROSPERO international prospective register of systematic reviews. The registration number is CRD42021247039. All data collections were presented in the supplementary file.

## Supplementary information

Supplementary information file

## Data Availability

For this systematic review we did not have access to the patient data from the original studies. We extracted information from the published articles of the included studies. The information extracted from the published articles of the included studies is available in this published article and its supplementary information files.
