# Supplementary material for: Systematic review of prognostic models in Parkinson’s disease
Source: NPJ Parkinsons Dis. 2025 Aug 29;11:266. doi: 10.1038/s41531-025-01112-x (PMC12397436; doi:10.1038/s41531-025-01112-x)
Supplement: Supplementary file 1 — Supplementary information file [file 41531_2025_1112_MOESM1_ESM.docx]

PRISMA checklist

| **Section and Topic** | **Item #** | **Checklist item** | **Location where item is reported** |
| --- | --- | --- | --- |
| **TITLE** | | |  |
| Title | 1 | Identify the report as a systematic review. | Page 1 |
| **ABSTRACT** | | |  |
| Abstract | 2 | See the PRISMA 2020 for Abstracts checklist. | Page 2 |
| **INTRODUCTION** | | |  |
| Rationale | 3 | Describe the rationale for the review in the context of existing knowledge. | Page 3 |
| Objectives | 4 | Provide an explicit statement of the objective(s) or question(s) the review addresses. | Page 3 |
| **METHODS** | | |  |
| Eligibility criteria | 5 | Specify the inclusion and exclusion criteria for the review and how studies were grouped for the syntheses. | Page 3 |
| Information sources | 6 | Specify all databases, registers, websites, organisations, reference lists and other sources searched or consulted to identify studies. Specify the date when each source was last searched or consulted. | Page 3 |
| Search strategy | 7 | Present the full search strategies for all databases, registers and websites, including any filters and limits used. | Supplementary Appendix 1 |
| Selection process | 8 | Specify the methods used to decide whether a study met the inclusion criteria of the review, including how many reviewers screened each record and each report retrieved, whether they worked independently, and if applicable, details of automation tools used in the process. | Page 3 |
| Data collection process | 9 | Specify the methods used to collect data from reports, including how many reviewers collected data from each report, whether they worked independently, any processes for obtaining or confirming data from study investigators, and if applicable, details of automation tools used in the process. | Page 3 and page 4 |
| Data items | 10a | List and define all outcomes for which data were sought. Specify whether all results that were compatible with each outcome domain in each study were sought (e.g. for all measures, time points, analyses), and if not, the methods used to decide which results to collect. | Page 4 |
|  | 10b | List and define all other variables for which data were sought (e.g. participant and intervention characteristics, funding sources). Describe any assumptions made about any missing or unclear information. | Page 4 |
| Study risk of bias assessment | 11 | Specify the methods used to assess risk of bias in the included studies, including details of the tool(s) used, how many reviewers assessed each study and whether they worked independently, and if applicable, details of automation tools used in the process. | Page 4 |
| Effect measures | 12 | Specify for each outcome the effect measure(s) (e.g. risk ratio, mean difference) used in the synthesis or presentation of results. | Page 4 |
| Synthesis methods | 13a | Describe the processes used to decide which studies were eligible for each synthesis (e.g. tabulating the study intervention characteristics and comparing against the planned groups for each synthesis (item #5)). | Page 4 |
|  | 13b | Describe any methods required to prepare the data for presentation or synthesis, such as handling of missing summary statistics, or data conversions. | Page 4 |
|  | 13c | Describe any methods used to tabulate or visually display results of individual studies and syntheses. | Page 4 |
|  | 13d | Describe any methods used to synthesize results and provide a rationale for the choice(s). If meta-analysis was performed, describe the model(s), method(s) to identify the presence and extent of statistical heterogeneity, and software package(s) used. | Page 4 |
|  | 13e | Describe any methods used to explore possible causes of heterogeneity among study results (e.g. subgroup analysis, meta-regression). | Page 4 |
|  | 13f | Describe any sensitivity analyses conducted to assess robustness of the synthesized results. | Page 4 |
| Reporting bias assessment | 14 | Describe any methods used to assess risk of bias due to missing results in a synthesis (arising from reporting biases). | Page 4 |
| Certainty assessment | 15 | Describe any methods used to assess certainty (or confidence) in the body of evidence for an outcome. | Page 4 |
| **RESULTS** | | |  |
| Study selection | 16a | Describe the results of the search and selection process, from the number of records identified in the search to the number of studies included in the review, ideally using a flow diagram. | Page 4 and figure 1 |
|  | 16b | Cite studies that might appear to meet the inclusion criteria, but which were excluded, and explain why they were excluded. | Figure 1 |
| Study characteristics | 17 | Cite each included study and present its characteristics. | Table 1 |
| Risk of bias in studies | 18 | Present assessments of risk of bias for each included study. | Table 3, supplementary table 9, 11,13, 14 |
| Results of individual studies | 19 | For all outcomes, present, for each study: (a) summary statistics for each group (where appropriate) and (b) an effect estimate and its precision (e.g. confidence/credible interval), ideally using structured tables or plots. | Page 4, table 1,2, supplementary table 7 |
| Results of syntheses | 20a | For each synthesis, briefly summarise the characteristics and risk of bias among contributing studies. | Page 6 and table 3, Supplementary table 8, 9, 10, 11,12,13,14 |
|  | 20b | Present results of all statistical syntheses conducted. If meta-analysis was done, present for each the summary estimate and its precision (e.g. confidence/credible interval) and measures of statistical heterogeneity. If comparing groups, describe the direction of the effect. | Page 5, table 2, supplementary table 1,2,3,4,5 |
|  | 20c | Present results of all investigations of possible causes of heterogeneity among study results. | Page 4 and 6, table 1, supplementary 8 |
|  | 20d | Present results of all sensitivity analyses conducted to assess the robustness of the synthesized results. | NA |
| Reporting biases | 21 | Present assessments of risk of bias due to missing results (arising from reporting biases) for each synthesis assessed. | Page 6, table 2, supplementary table 3,4 |
| Certainty of evidence | 22 | Present assessments of certainty (or confidence) in the body of evidence for each outcome assessed. | Page 6, table 2, supplementary table 7 |
| **DISCUSSION** | | |  |
| Discussion | 23a | Provide a general interpretation of the results in the context of other evidence. | Page 6 and 7 |
|  | 23b | Discuss any limitations of the evidence included in the review. | Page 8 |
|  | 23c | Discuss any limitations of the review processes used. | Page 8 |
|  | 23d | Discuss implications of the results for practice, policy, and future research. | Page 8 |
| **OTHER INFORMATION** | | |  |
| Registration and protocol | 24a | Provide registration information for the review, including register name and registration number, or state that the review was not registered. | Page 4 |
|  | 24b | Indicate where the review protocol can be accessed, or state that a protocol was not prepared. | Page 4 |
|  | 24c | Describe and explain any amendments to information provided at registration or in the protocol. | Page 4 |
| Support | 25 | Describe sources of financial or non-financial support for the review, and the role of the funders or sponsors in the review. | Page 9 |
| Competing interests | 26 | Declare any competing interests of review authors. | Page 9 |
| Availability of data, code and other materials | 27 | Report which of the following are publicly available and where they can be found: template data collection forms; data extracted from included studies; data used for all analyses; analytic code; any other materials used in the review. | Page 4 |

*From:*  Page MJ, McKenzie JE, Bossuyt PM, Boutron I, Hoffmann TC, Mulrow CD, et al. The PRISMA 2020 statement: an updated guideline for reporting systematic reviews. BMJ 2021;372:n71. doi: 10.1136/bmj.n71

Supplementary Appendix 1

The electronic search strategies were as follows, including the numbers of results for each search string on 20/02/2021:

MEDLINE:

| 1. Parkinson Disease/ | 68588 |
| --- | --- |
| 2. parkinson$.tw. | 122016 |
| 3. 1 or 2 | 129752 |
| 4. ((prognos$ or predict or prediction$) adj6 (model$ or tool$ or risk$ or probabilit$ or rule$ or aid$ or index$)).tw. | 201452 |
| 5. 3 and 4 | 585 |
| 6. exp animals/ not humans.sh. | 4790230 |
| 7. 5 not 6 | 560 |

EMBASE:

| 1. Parkinson Disease/ | 160781 |
| --- | --- |
| 2. parkinson$.tw. | 173166 |
| 3. 1 or 2 | 205088 |
| 4. ((prognos$ or predict or prediction$) adj6 (model$ or tool$ or risk$ or probabilit$ or rule$ or aid$ or index$)).tw. | 282833 |
| 5. 3 and 4 | 1114 |
| 6. animal experiment/ not (human experiment/ or human/) | 2319665 |
| 7. 5 not 6 | 1087 |

Supplementary table 1. Prognostic factors in studies

| **First author/Year** | **Prognostic factors** | **Definition and method for measurement of candidate predictors** | **Handling of predictors** | **Outcome(s)** |
| --- | --- | --- | --- | --- |
| Almeida 2016 ^7^ | Demographic (7 predictors), PD severity (17 predictors), PD-specific medications (5 predictors), Non-PD specific medications (10 predictors), Disability (2 predictors), Balance/Mobility (4 predictors), Self-efficacy (2 predictors) | PD disease severity (UPDRS motor section, H&Y stage). Disability (UPDRS ADL, S&E scale). FOG (≥1 point on item 14 of UPDRS ADL). Health-related quality of life (8 item PDQ-39). Balance confidence (ABS, FES-I). Performance-based balance (BBS, FRT, TUG, DGI). | All continuous variables were dichotomised | Recurrent falls |
| Ashburn 2001 ^8^ | Age, Sex, H&Y, UPDRS motor section, SAS, Total drugs, Number of falls, Near falls, Worried about falls, Leeds anxiety scale, Leeds depression scale | Severity of motor impairment (UPDRS motor section, H&Y stage). Anxiety and depression (Leeds Inventory). Balance performance (TUG). Dynamic balance control (FRT). | Continuous variables kept in continuous | Falls |
| Custodio 2016 ^9^ | Sex, Age, PD duration, UPDRS part 3, Dyskinesia scale, Abnormal axial posture, Freezing, FOG, ACE, IFS, PFAQ, GDS, Number of falls, Fear of falling, Physical activity | Fear of falling (FES-I). FOG (FOG-Q). Disease severity (UPDRS III). Neuropsychological testing (ACE, IFS and GDS). | Continuous variables kept in continuous | Falls |
| Duncan 2015 ^10^ | Fall in previous 12-month, FOG in the past month, Self-selected gait speed <1.1m/s | Fall history (none, once,2-10 times, weekly, daily). FOG (FOG-Q). Self-selected gait speed<1.1 m/s was determined using the mean of two gait speed trails collected during a 10-m walk. | All variables were dichotomised | Falls |
| Ehgoetz Martens 2018 ^11^ | Tremor dominant/non tremor dominant ratio, FOG-Q total, UPDRS-III, TMT-A and TMT-B, DS-Forwards, DS-Backwards, LM-II, HADS-A and HADS-D, RBDSQ4, MMSE, Age, Disease duration at baseline | Motor symptoms (UPDRS III). Global gait disturbance (FOG-Q). Cognition (DS-Forwards, DS-Backwards, MMSE, LM-I, LM-II, TMT-A, TMT-B). Anxiety and depression (HADS-A and HADS-D). RBD (RBDSQ). | Continuous variables kept in continuous | Freezing of gait |
| Exarchos 2012 ^12^ | Medical history (12 predictors), Examinations (17 predictors), Medication (10 predictors) | Tremor, rigidity, bradykinesia used UPDRS score to define severity. | Variables were dichotomised or category, only age was left as continuous | Multiple symptoms and signs |
| Gervasoni 2015 ^13^ | Sex, Age, Syndrome, Assistive device, Disease duration, History of falls, UPDRS, GABS, LAPAQ, H&Y, Barthel index, GABS score (ithem6), GABS (item 7), Factor1 (Medial–lateral mean body sway and area), Factor2 (body sway velocity), Factor3 (length) | The physical examination (GABS). Total time spent on physical activities (LAPAQ). Factors were computed by PCA. | Most left continuous; one variable categorised and one variable dichotomised. | Falls, Recurrent falls |
| Gu 2020 ^14^ | Age, Age at onset, Sex, Side of symptom onset, duration, History of depression, History of malignancy, MDS-UPDRS II, MDS-UPDRS III, MOCA, Modified S&E ADL, SCOPA-AUT, RBDQ and STAI. Total tau, t-tau, phosphorylated tau 181p, Aβ1-42 and α-synuclein. Mean caudate, Mean Putaminal, Caudate asymmetry, Putaminal asymmetry | Not stated | Continuous variables left as continuous, only H&Y stage was viewed as category. | Depression |
| Kelly 2019 ^15^ | Age, Levodopa dose (mg) per weight (kg), UPDRS Part II score, Sex | Age is at levodopa commencement, levodopa dose and weight are both at first visit after levodopa commencement, MDS-UPDRS II is prior to levodopa commencement. | Continuous variables kept in continuous | Dyskinesia |
| Kerr 2010 ^16^ | Body bradykinesia, Postural stability, Gait, Posture, Rising from chair, Leg agility, Rapid alternating tasks, Hand movements, Finger taps, Rigidity, Action or postural tremor, Resting tremor, Expression, Speech | Balance, gait, and falls risk (Tinetti, BBS, TUG, FRT, PPA). | Continuous variables kept in continuous | Falls |
| Lindholm 2016 ^17^ | History of falls in 12 months, History of near falls, History of FOG, Gait speed, NRT, UPDRS item 30, and Abnormal tandem gait. | History of FOG (FOG-Q), gait speed measurement was by the 10-meter walk test. | All variables were dichotomised | Falls |
| Liu 2017 ^18^ | MDS-UPDRS part III, H&Y, Age at onset, baseline MMSE, years of education, sex, depression, and GBA mutation status | Not stated | Continuous variables kept in continuous | Global cognitive impairment and dementia within 10 years from onset |
| Lo 2019 ^19^ | Smartphone features, details not specified | Not stated | Unclear | Falls, freezing of gait, postural instability, difficulty doing hobbies, cognitive impairment, dependency |
| Macleod 2018 ^20^ | Age, Sex, Severity of bradykinesia, H&Y, Severity of axial features, MMSE, CCI score, Pack years of smoking | Severity of bradykinesia, axial features (UPDRS III). Dependency (S&E at follow-up visits and defined using a cut-off of<80%). | Continuous variables kept in continuous | Mortality, Dependency, Death/dependency |
| Mak 2014 ^21^ | Age, Sex, Duration of PD, Fall history, Geriatric Depression Scale, H&Y score, UPDRS III, Levodopa dosage, PASE, FOGQ, Mini-BESTest score, ABC score, MDRS-IP score | Disease severity, PD-specific motor impairment, disability (H&Y, UPDRS-III). Depressive symptoms (Chinese version of the short-form Geriatric Depression Scale). Physical activity level (PASE). Freezing on their gait performance (FOG-Q). Balance performance (Mini-BESTest). Fear of falling (Chinese version of ABC). Executive function (MDRS-IP). | Continuous variables kept in continuous | Recurrent falls |
| Paul 2013 ^22^ | Sex, Age, Physical activity, Number of falls in previous 12 months, Fear of falling score, PD duration, UPDRS motor score, Dyskinesia score, Abnormal axial posture, FOG, severity-FOGQ score, MMSE score, FAB score, Knee extensor strength, Functional Reach, Near tandem stand with eyes shut, Alternate step, Five times sit-to-stand, Fast gait speed, Self-selected gait speed, Standing balance with narrow base of support, Sway on floor, Sway on foam, Maximal balance, Coordinated stability | Fear of falling (FES-I). PD severity, dyskinesia, posture (UPDRS). FOG (FOG-Q). | Most continuous kept as continuous variables, only gait speed cut as dichotomous | Falls |
| Phongpreecha 2020 ^23^ | Global cognitive, Learning & memory, Attention/working memory, Verbal fluency, MDS-UPDRS part III, H&Y scale, Age, Education, Sex, Disease duration, Total LEDD, GDS-15. APOE rs429358 and rs7412 and MAPT rs1800547 and APOE genotype and E326K polymorphism | Global cognitive (MoCA). Learning & memory (HVLT-R). Attention/working memory (WAIS, TMT). Verbal fluency (animals and letters F-A-S). Visuospatial (JoLO). Severity of motor symptoms (MDS-UPDRS, Modified H&Y scale). | Unclear | Normal cognition, Mild cognitive impairment,  Dementia |
| Pouwels 2013 ^24^ | Age, Sex, BMI, Smoking status, Fracture, Asthma/COPD, rheumatoid arthritis, Hyperthyroidism, Hypothyroidism, Renal disease, Cancer, Congestive heart failure, Cerebrovascular disease, Diabetes mellitus, Inflammatory bowel disease, dementia. A prescription in the previous 6 months for CNS medication (10 predictors) | The chronic diseases were identified with CPRD records which described the presence of the disease. | Age was kept as continuous, BMI was cut in 4 groups, other variables were dichotomous or category. | Osteoporosis, Hip fracture risks |
| Redensek 2019 ^25^ | Sex, Age, tremor-predominant PD or other, Body side of disease initiation, RBD, Depression, Constipation, Olfactory dysfunction, Time from diagnosis to initiation of levodopa treatment, Beta-blockers, Non-steroidal anti-inflammatory drugs, Calcium channel blockers, Statins, Tobacco smoking, Alcohol consumption, Coffee consumption. 34 single nucleotide polymorphisms. | Not stated | Continuous variables kept in continuous | Motor fluctuations, Dyskinesia |
| Schapira 2012 ^26^ | Age, Levodopa dose (mg) per weight (kg), UPDRS Part II score, Sex | Definition clear from description of variables | Continuous variables kept in continuous | Dyskinesias |
| Schrag 2017 ^27^ | Age, Sex, years of Education, Disease duration, Sense of smell, RBD, Depression, Parkinson’s disease severity, Tremor-dominant subtype, Postural instability and gait difficulty subtype, Indeterminate motor subtype, APOE ε4 status, Mean putaminal uptake, Mean caudate uptake, Putaminal asymmetry, Caudate asymmetry, Aβ42,α-synuclein, α-synuclein, Total tau, Phosphorylated tau181, Total protein | Sense of smell (UPSIT). RBD (RBD-Q). Depression (Geriatric Depression Scale). Parkinson's disease severity (MDS-UPDRS). | Continuous variables kept in continuous | Cognitive impairment |
| Velseboer 2016 ^28^ | Age, Sex, Symptom duration, H&Y, UPDRS tremor score, UPDRS rigidity score, UPDRS bradykinesia score, UPDRS axial score, Levodaopa Equivalent dosage, Number of comorbid organ systems, presence of depression, COWAT score, Animal fluency score, MMSE score | Motor impairment (UPDRS). Number of comorbid organ systems (CIRS). Presence of depressive symptoms (HADS). Semantic fluency was assessed with the animal fluency test. | Continuous variables kept in continuous | Composite outcome (instability, dementia, or death) |
| Wang 2017 ^29^ | Baseline disease duration, baseline age, treatment (active deprenyl only), time, and the interaction term of treatment and time | Not stated | Unclear | Need for levodopa treatment |
| Wang 2017 ^30^ | Time (since baseline visit), age, Sex, education, Duration of illness, HAMD, MOCA, LEDD | Depression (HAMD). Global cognitive (MOCA). Duration of illness was defined as the time since PD diagnosis by a medical professional. | Continuous variables left as continuous, only H&Y stage was viewed as category. | Imbalance |
| Ye 2017 ^31^ | Age, Sex, Education and 25 predictors of neuropsychological test. Neuropsychological test: 3 predictors in attention, 4 predictors in language and related function, 2 in visuospatial function, 3 in verbal-memory function, 3 in visual-memory function, 10 in frontal/executive function. | Neuropsychological evaluation (SNSB). | Continuous variables were cut off as dichotomised, only age kept as continuous. | Dementia |
| Abbreviation: ABC=Activities-specific Balance Confidence; ABS=Activities-specific Balance Confidence Scale; ACE=Addenbrooke's Cognitive Examination; ADL=activities of daily living; Mini-BESTest=Mini Balance Evaluation Systems Test; APOE=apolipoprotein E gene; BBS=Berg Balance Scale; BMI=Body mass index; CCI=Charlson comorbidity index; CIRS= Cumulative Illness Rating Scale; CNS=central nervous system; COPD= chronic obstructive pulmonary disease; COWAT=Controlled Oral Word Association Test; CPRD=Clinical Practice Research Datalink; CPRD=Clinical Practice Research Datalink; DGI=Dynamic Gait Index; DS=Digit Span; FBA=Frontal Assessment Battery; FES-I=Falls Efficacy Scale-International; FOG= freezing of gait; FOG-Q=Freezing of Gait Questionnaire; FRT=Functional Reach Test; GABS=Gait and Balance Scale; GBA= β-glucocerebrosidase; GDS=Global Deterioration Scale; H&Y=Hoehn and Yahr; HADS=Hospital Anxiety and Depression Scale; HAMD=Hamilton Depression Rating Scale; HVLT-R=Hopkins Verbal Learning Test-Revised; IFS=INECO Frontal Screening; JoLO=Benton Judgment of Line Orientation; LAPAQ=LASA physical activity questionnaire; LEDD=levodopa equivalent daily dose; LM=Logical Memory; MAPT=microtubule-associated protein tau gene; MDRS-IP=Mattis Dementia Rating Scale Initiation/Perseveration subset; MDS-UPDRS=Movement Disorder Society Revision of the Unified Parkinson Disease Rating Scale; MMSE=Mini Mental State Examination; MOCA= Montreal Cognitive Assessment; NRT= Nutt Retropulsion test; PASE=Physical Activity Scale for the Elderly; PCA=principal component analysis; PD=Parkinson’s disease; PDQ-39=Parkinson’s Disease Questionnaire; PFAQ= Pfeffer Functional Activities Questionnaire; PPA=Physiological Profile Assessment; RBD=Rapid eye movement sleep behaviour disorder; RBDQ=Rapid eye movement sleep behaviour disorder questionnaire; S&E=Schwab and England; SAS=Self-Assessed Disability Scale; SCOPA-AUT= Scales for Outcomes in Parkinson's Disease Autonomic Questionnaire; SNSB=Seoul Neuropsychological Screening Battery; STAI=State Trait Anxiety Inventory score; TMT=Trail making Test; TUG=Time Up & Go Test; UPDRS=Unified Parkinson Disease Rating Scale; UPSIT=University of Pennsylvania Smell Identification Test; WAIS= Wechsler Adult Intelligence Scale | | | | |

Supplementary table 2. Information on prognostic factors in the final models by study

| **First author/Year** | **Prognostic factors in final model** | **Outcome(s)** |
| --- | --- | --- |
| Almeida 2016 ^7^ | Model 1: Falls in the past year, motor fluctuations, UPDRS ADL, LED model 2 add Berg balance scale | Recurrent falls |
| Almeida 2016 ^7^ | Model 2: Falls in the past year, motor fluctuations, UPDRS ADL, LED, BBS | Recurrent falls |
| Ashburn 2001 ^8^ | Number of falls, Worried about falls, Leeds anxiety scale | Falls |
| Custodio 2016 ^9^ | PD duration, ACE, Number of falls, Physical activity, FOG (score) | Falls |
| Duncan 2015 ^10^ | Fall in previous 12-month, FOG in the past month, Self-selected gait speed <1.1m/s | Falls |
| Ehgoetz Martens 2018 ^11^ | Age, Disease duration at baseline, Tremor dominant/non tremor dominant ratio, FOG-Q total, TMT-B, HADS-A and HADS-D (2 models with the same prognostic factors) | Freezing of gait |
| Exarchos 2012 ^12^ | Medical history (12 predictors), Examinations (17 predictors), Medication (10 predictors) | Multiple symptoms and signs |
| Gervasoni 2015 ^13^ | Model 1 (Falls): Sex, Age, Syndrome, Assistive device, Disease duration, History of falls, UPDRS, GABS, LAPAQ, H&Y, Barthel index, GABS score (ithem6), GABS (item 7), Factor1 (Medial–lateral mean body sway and area), Factor2 (body sway velocity), Factor3 (length) | Falls |
| Gervasoni 2015 ^13^ | Model 2 (Recurrent falls): Sex, Age, Syndrome, Assistive device, Disease duration, History of falls, UPDRS, GABS, LAPAQ, H&Y, Barthel index, GABS score (ithem6), GABS (item 7), Factor1 (Medial–lateral mean body sway and area), Factor2 (body sway velocity), Factor3 (length) | Recurrent falls |
| Gu 2020 ^14^ | Age, Age at onset, History of depression, History of malignancy, MDS-UPDRS II, MDS-UPDRS III, Modified S&E ADL, SCOPA-AU, RBDQ, phosphorylated tau, Total tau: Aβ1-42 ratio | Depression |
| Kelly 2019 ^15^ | Age, Levodopa dose (mg) per weight (kg), UPDRS Part II score, Sex | Dyskinesia |
| Kerr 2010 ^16^ | Body bradykinesia, Postural stability, Gait, Posture, Rising from chair, Leg agility, Rapid alternating tasks, Hand movements, Finger taps, Rigidity, Action or postural tremor, Resting tremor, Expression, Speech | Falls |
| Lindholm 2016 ^17^ | Model 1: History of falls in 12 months, History of FOG, Gait speed | Falls |
| Lindholm 2016 ^17^ | Model 2: History of falls in 12 months, History of near falls, History of FOG, Gait speed, NRT, UPDRS item 30, and Abnormal tandem gait. | Falls |
| Liu 2017 ^18^ | MDS-UPDRS part III, H&Y, Age at onset, age, baseline MMSE, years of education, sex, depression, and GBA mutation status | Global cognitive impairment and dementia within 10 years from onset |
| Lo 2019 ^19^ | Model 1-6: Smartphone features, details not specified | Falls, freezing of gait, postural instability, difficulty doing hobbies, cognitive impairment, dependency |
| Macleod 2018 ^20^ | Model 1 (all-cause mortality) Age, Sex, Severity of axial features, CCI (effect in first 4 years of follow-up), CCI (effect after 4 years) | Mortality |
| Macleod 2018 ^20^ | Model 2 (all-cause mortality) Age, Sex, Severity of axial features | Mortality |
| Macleod 2018 ^20^ | Model 3 (functional dependency) Age, Smoking history, Severity of axial features, MMSE score | Dependency |
| Macleod 2018 ^20^ | Model 4 (death or dependency) Age, Smoking history, Severity of axial features, MMSE score | Death/dependency |
| Mak 2014 ^21^ | Fall history, ABC score, MDRS-IP score | Recurrent falls |
| Paul 2013 ^22^ | Number of falls in previous 12 months, FOG in past month, Self-selected gait speed | Falls |
| Phongpreecha 2020 ^23^ | Global cognitive, Learning & memory, Attention/working memory, Verbal fluency, MDS-UPDRS part III, H&Y scale, Age, Years of education, Sex, Disease duration, Total LEDD, GDS-15. APOE rs429358 and rs7412 and MAPT rs1800547 and APOE genotype and E326K polymorphism | Normal cognition, Mild cognitive impairment,  Dementia |
| Pouwels 2013 ^24^ | Model 1 (Osteoporotic fracture): Age, sex, current smoking, a BMI below 20, a BMI of 30 or more, a history of fracture, the use of oral glucocorticoids in the previous 6 months and a history of RA, history of antidepressant use in the previous 6 months, history of renal disease and dementia and a history of falling | Osteoporosis |
| Pouwels 2013 ^24^ | Model 2 (Hip fracture): Age, sex, current smoking, a BMI below 20, a BMI of 30 or more, a history of fracture, the use of oral glucocorticoids in the previous 6 months and a history of RA, history of antidepressant use in the previous 6 months | Hip fracture risks |
| Redensek 2019 ^25^ | Model 1 (Motor fluctuations) Age, Time from diagnosis to initiation of levodopa treatment, Tobacco smoking | Motor fluctuations |
| Redensek 2019 ^25^ | Model 2 (Motor fluctuations) Age, Time from diagnosis to initiation of levodopa treatment, Tobacco smoking, COMT rs165815, DRD3 rs6280, BIRC5 rs9904341 | Motor fluctuations |
| Redensek 2019 ^25^ | Model 3 (Dyskinesia) Sex, age, tremor-predominant PD, Beta-blockers, alcohol consumption, Time from diagnosis to initiation of levodopa treatment | Dyskinesia |
| Redensek 2019 ^25^ | Model 4 (Dyskinesia) Sex, age, tremor-predominant PD, Beta-blockers, alcohol consumption, Time from diagnosis to initiation of levodopa treatment, CAT rs1001179, SOD2 rs4880, NOS1 rs2293054, COMT rs165815, SLC22A1 rs628031 | Dyskinesia |
| Schapira 2012 ^26^ | Age, Levodopa dose (mg) per weight (kg), UPDRS Part II score, Sex | Dyskinesias |
| Schrag 2017 ^27^ | Age, RBD, Mean caudate uptake, CSF Aβ42, UPSIT | Cognitive impairment |
| Velseboer 2016 ^28^ | Age, UPDRS axial score, Animal fluency score | Composite outcome (instability, dementia, or death) |
| Wang 2017 ^29^ | Baseline disease duration, baseline age, treatment (active deprenyl only), time, and the interaction term of treatment and time | Need for levodopa treatment |
| Wang 2017 ^30^ | Time (since baseline visit), age, Sex, education, Duration of illness, HAMD, MOCA, LEDD | Imbalance |
| Ye 2017 ^31^ | Age, Sex, Education, COWAT supermarket, K-BNT, Go-no-go test, Stroop colour reading. | Dementia |

Abbreviation: ADL=activities of daily living; APOE=apolipoprotein E gene; BBS=Berg Balance Scale; BMI=Body mass index; CCI=Charlson comorbidity index; COWAT=Controlled Oral Word Association Test; FOG= freezing of gait; FOG-Q=Freezing of Gait Questionnaire; GABS=Gait and Balance Scale; GBA= β-glucocerebrosidase; GDS=Global Deterioration Scale; H&Y=Hoehn and Yahr; HADS=Hospital Anxiety and Depression Scale; HAMD=Hamilton Depression Rating Scale; K-BNT=Korean-Boston Naming Test; LAPAQ=LASA physical activity questionnaire; LEDD=levodopa equivalent daily dose; MAPT=microtubule-associated protein tau gene; MDRS-IP=Mattis Dementia Rating Scale Initiation/Perseveration subset; MDS-UPDRS=Movement Disorder Society Revision of the Unified Parkinson Disease Rating Scale; MMSE=Mini Mental State Examination; MOCA= Montreal Cognitive Assessment; NRT= Nutt Retropulsion test; PASE=Physical Activity Scale for the Elderly; PD=Parkinson’s disease; RBD=Rapid eye movement sleep behaviour disorder; RBDQ=Rapid eye movement sleep behaviour disorder questionnaire; S&E=Schwab and England; SCOPA-AUT= Scales for Outcomes in Parkinson's Disease Autonomic Questionnaire; TMT=Trail making Test; UPDRS=Unified Parkinson Disease Rating Scale; UPSIT=University of Pennsylvania Smell Identification Test

Supplementary table 3. Follow-up information

| **First author/Year** | **Number lost to follow-up** | **Sample size^a^** | **Time of outcome occurrence or summary of duration of follow-up** |
| --- | --- | --- | --- |
| Almeida 2016 ^7^ | 4 | Models 1&2: 229 | 12 months |
| Ashburn 2001 ^8^ | 6 | 63 | 3 months |
| Custodio 2016 ^9^ | Not stated | 59 | 1 year |
| Duncan 2015 ^10^ | Not stated | 171 | 6 months |
| Ehgoetz Martens 2018 ^11^ | Not stated | Model 1: 117  Model 2: 75 | 6-24 months |
| Exarchos 2012 ^12^ | Not stated | 230 | No information |
| Gervasoni 2015 ^13^ | 7 | 53 | 6 months |
| Gu 2020 ^14^ | 57 | 312 | 2 years |
| Kelly 2019 ^15^ | Not stated | 62 | 200 weeks |
| Kerr 2010 ^16^ | 5 | 130 | 6 months |
| Lindholm 2016 ^17^ | 5 | 135 | 6 months |
| Liu 2017 ^18^ | Not stated | 1350 (dev); 1132 (val) | 10 years |
| Lo 2019 ^19^ | 17 (fall),6 (freezing of gait), 31 (postural instability), 30 (cognitive impairment), 10 (difficulty doing hobbies), 7 (functional impairment/need for help at home) | 237 | 18 months |
| Macleod 2018 ^20^ | Not stated | Models 1&2: 198(dev); 192(val)  Model 3: 176(dev); 162(val)  Model 4: 176(dev); 162(val) | Up to 12 years from diagnosis (dev); up to 8 years from diagnosis (val) |
| Mak 2014 ^21^ | 3 | 144 | 12 months |
| Paul 2013 ^22^ | Not stated | 205 | 6 months |
| Phongpreecha 2020 ^23^ | 313 | 827 | 1-2 years |
| Pouwels 2013 ^24^ | Not stated | Models 1&2: 4411 | Average 4 years |
| Redensek 2019 ^25^ | Not stated | Models 1&2 :231  Models 3&4: 231 | Median follow-up time: 7.1 years (motor fluctuations), 6.6 years (dyskinesia) |
| Schapira 2012 ^26^ | Not stated | NS | 134-208 weeks |
| Schrag 2017 ^27^ | 76 | 390 | 2 years |
| Velseboer 2016 ^28^ | 5 (dev), 7 (val) | 111(dev); 108(val) | 5 years |
| Wang 2017 ^29^ | Not stated | 755 | 24 months |
| Wang 2017 ^30^ | 24 | 76 | 36 months |
| Ye 2017 ^31^ | Not stated | 216 | Mean follow-up time:2.7 years |

^a^Initial numbers, where present, designate multiple models in one paper

Abbreviation: dev=development; val=validation.

Supplementary table 4. Details information of missing data

| **First author/Year** | **Number of participants with any missing value (predictors and outcomes)** | **Number of participants with missing data for each predictor** |
| --- | --- | --- |
| Almeida 2016 ^7^ | 4 | Not stated |
| Ashburn 2001 ^8^ | 4 | Not stated |
| Custodio 2016 ^9^ | Not stated | Not stated |
| Duncan 2015 ^10^ | 0 | 0 |
| Ehgoetz Martens 2018 ^11^ | Not stated | Not stated |
| Exarchos 2012 ^12^ | Not stated | Not stated |
| Gervasoni 2015 ^13^ | 7 | Not stated |
| Gu 2020 ^14^ | 57 | Not stated |
| Kelly 2019 ^15^ | Not stated | Not stated |
| Kerr 2010 ^16^ | Not stated | Not stated |
| Lindholm 2016 ^17^ | Not stated | Not stated |
| Liu 2017 ^18^ | 348 | Not stated |
| Lo 2019 ^19^ | 24 (fall), 7 (freezing of gait), 32 (postural instability), 32 (cognitive impairment), 11 (difficulty doing hobbies), 177 (functional impairment/need for help at home) | Not stated |
| Macleod 2018 ^20^ | No missing data | Not applicable |
| Mak 2014 ^21^ | Not stated | Not stated |
| Paul 2013 ^22^ | Not stated | Not stated |
| Phongpreecha 2020 ^23^ | 2% of the total observed features | Not stated |
| Pouwels 2013 ^24^ | Not stated | Not stated |
| Redensek 2019 ^25^ | 11 | Not stated |
| Schapira 2012 ^26^ | Not stated | Not stated |
| Schrag 2017 ^27^ | Unclear | 2 (GDS), 34 (RBDSQ), 37 (APOE status). Baseline CSF data were missing for Aβ42 and α-synuclein in 10 patients, for p-tau in 12 patients, for t-tau in 14 patients, and for total protein in 31 patients. |
| Velseboer 2016 ^28^ | Development dataset: 10 missing predictors | 9 (fluency tests), 1 (depression rating) |
| Wang 2017 ^29^ | 45 | 44 (disease duration recorded), 1 (UPDRS) |
| Wang 2017 ^30^ | 2 | 2 (MDS-UPDRS) |
| Ye 2017 ^31^ | Not stated | Not stated |

Abbreviation: GDS=Geriatric Depression Scale; MDS-UPDRS=Movement Disorder Society Revision of the Unified Parkinson Disease Rating Scale; RBDQ=Rapid eye movement sleep behaviour disorder questionnaire; UPDRS=Unified Parkinson Disease Rating Scale

Supplementary table 5. Details on model development

| **First author/Year** | **Modelling assumption satisfied** | **Method for selection of predictors for inclusion in multivariable modelling** | **Method for selection of predictors during multivariable modelling** |
| --- | --- | --- | --- |
| Almeida 2016 ^7^ | Yes | p<0.1 in univariable analysis | Backward stepwise |
| Ashburn 2001 ^8^ | No Information | All available variables in the study | Forward and backwards selection |
| Custodio 2016 ^9^ | No Information | Univariable analysis | BIC and AIC select predictors |
| Duncan 2015 ^10^ | Yes | Same predictors as previous published model does not need to select variables | Not stated |
| Ehgoetz Martens 2018 ^11^ | No Information | Based on previous research | forward stepwise (p=0.05) and backward stepwise (p=0.1) |
| Exarchos 2012 ^12^ | No Information | Not stated | Wrapper feature selection |
| Gervasoni 2015 ^13^ | No Information | Univariate logistic models with p<0.1 | Variables in univariate logistic models with p<0.1 entered model |
| Gu 2020 ^14^ | No Information | All variables entered (XGBoost). Univariate logistics model with p<0.1 (Logistic regression) | XGBoost (XGBoost). Forward and backwards selection (Logistic regression) |
| Kelly 2019 ^15^ | No Information | External validation does not need to select variables | Not applicable |
| Kerr 2010 ^16^ | No Information | p<0.01 in univariable analysis (Independent sample t tests, chi-squared test) | Unclear |
| Lindholm 2016 ^17^ | No Information | Same predictors as previous published model does not need to select variables (model1). Based on previous research (model2). | Used same predictors as previous study, pseudo-external validation (model1). Backward stepwise (model2) |
| Liu 2017 ^18^ | Yes | Excluded the predictor that violated the PH assumption. | Backward selection using AIC |
| Lo 2019 ^19^ | Not Applicable | All available variables in the study | Models developed with all 998 features and top 30 smartphone features |
| Macleod 2018 ^20^ | Yes | Univariable analysis p<0.2 | Backward stepwise regression |
| Mak 2014 ^21^ | No Information | All available variables in the study | Significant predictors (p<0.5) from full model included in final model |
| Paul 2013 ^22^ | No Information | Univariate logistics regression p<0.1 for continuous variable and highly correlated (r>=0.7) variables were not entered into the multivariate model. | Bootstrap with backward stepwise |
| Phongpreecha 2020 ^23^ | No Information | Not stated | Not stated |
| Pouwels 2013 ^24^ | No Information | All available variables in the study | Forward selection with p<0.05 |
| Redensek 2019 ^25^ | No Information | Univariate cox regression | Lasso penalization |
| Schapira 2012 ^26^ | No Information | Not stated | Not stated |
| Schrag 2017 ^27^ | Yes | Univariate logistics model < 0.05 | Not highly correlated (r>0·5) and were significantly different between those with and those without cognitive impairment (p≤0·05) |
| Velseboer 2016 ^28^ | No Information | All available variables in the study | Stepwise backward selection based on AIC, Candidate predictors that appeared in 50% or more of the multivariable models from the different imputed datasets were retained in the final model |
| Wang 2017 ^29^ | No Information | Not stated | Not stated |
| Wang 2017 ^30^ | Yes | All available variables in the study | Not stated |
| Ye 2017 ^31^ | No Information | Neuropsychological tests with significant p values (<0.05) and a moderate effect size (HR >2) in the univariate analyses. | Not stated |

Supplementary table 6. Methods of model validation

| **First author/Year** | **Internal validation** | **External validation** | **Updated after external validation** | **Model type** |
| --- | --- | --- | --- | --- |
| Almeida 2016 ^7^ | 1000 bootstrap | Not applicable | No | Cox regression |
| Ashburn 2001 ^8^ | No information | Not applicable | No | Logistics regression |
| Custodio 2016 ^9^ | No information | Not applicable | No | Logistics regression |
| Duncan 2015 ^10^ | No information | Geographical (No true external validation) | No | Logistic regression |
| Ehgoetz Martens 2018 ^11^ | No information | Not applicable | No | Logistics regression |
| Exarchos 2012 ^12^ | 10-fold cross validation | Not applicable | No | Partial decision tree |
| Gervasoni 2015 ^13^ | Leave-one-out cross-validation | Not applicable | No | Univariable logistics regression; Logistics regression |
| Gu 2020 ^14^ | 70% training and 30% testing (XGBoost); 1000 bootstrap (Logistics regression) | Not applicable | No | XGBoost; Logistics regression |
| Kelly 2019 ^15^ (external model validation study) | Not applicable | Geographical | No | Cox regression |
| Kerr 2010 ^16^ | Leave-one-out cross-validation | Not applicable | No | Logistics regression |
| Lindholm 2016 ^17^ | No information (No true external validation); No information (development model) | Geographical (No true external validation); Not applicable (development model) | No | Logistics regression |
| Liu 2017 ^18^ | 10000 randomly resampled subsets | The external validation in other three cohorts (1132 subjects) | No | Frailty Cox regression |
| Lo 2019 ^19^ | 10-fold and subject-wise cross validation schemes and leave-one-out cross validation | Not applicable | No | Random forest |
| Macleod 2018 ^20^ | 500 bootstraps | Geographical in ParkWest study (Norway, 192 patients) | Yes | Parametric Proportional hazards model (Weibull) |
| Mak 2014 ^21^ | No information | Not applicable | No | Logistics regression |
| Paul 2013 ^22^ | 1000 bootstrap | Not applicable | No | Logistics regression |
| Phongpreecha 2020 ^23^ | 100 iterations of two-layered cross-validations | Not applicable | No | Generalized multitask models |
| Pouwels 2013 ^24^ | 10-fold cross-validation | Not applicable | No | Cox regression |
| Redensek 2019 ^25^ | Cross-validation | Not applicable | No | Cox regression with LASSO penalization |
| Schapira 2012 ^26^ | No information | Not applicable | No | Cox regression |
| Schrag 2017 ^27^ | 1000 bootstrap,10-fold cross-validation and data split (7:3) | Not applicable | No | Logistics regression |
| Velseboer 2016 ^28^ | 1000 bootstrap | Geographical in CamPalGN study (UK, 108 patients) | Yes | Logistics regression |
| Wang 2017 ^29^ | 5-fold cross-validation | Not applicable | No | Joint model – semiparametric multilevel latent trait model for multiple longitudinal outcomes and a survival submodel for event time |
| Wang 2017 ^30^ | Random split | Not applicable | No | Bayesian multivariate generalized linear mixed-effect model |
| Ye 2017 ^31^ | 1000 bootstrapping | Not applicable | No | Cox regression |

Supplementary table 7. Model performance

| **First author/Year** | **Calibration and discrimination performance** | **Classification measures and a-priori cut points** |
| --- | --- | --- |
| Almeida 2016 ^7^ | NA; AUC=0.84 (0.78-0.90) (model1); AUC=0.86 (0.81-0.92) (model2) | No information |
| Ashburn 2001 ^8^ | NA; NA | ROC curves. Cut off = two or more falls (model driven). Sensitivity = 0.86 (0.67-0.96), specificity = 0.86 (0.71-0.94) |
| Custodio 2016 ^9^ | Hosmer-Lemeshow test p-value=0.87; AUC = 0.93 | No information |
| Duncan 2015 ^10^ | Hosmer-Lemeshow test p-value=0.77; AUC = 0.83 (0.76,0.89) | Sensitivity = 0.91 (0.81-0.97), specificity = 0.66 (0.56-0.75) |
| Ehgoetz Martens 2018 ^11^ | Hosmer-Lemeshow test p-value=0.56; NA | Overall predictive success of 82.1% |
| Exarchos 2012 ^12^ | NA; NA | Sensitivity = 0.52-0.88, specificity = 0.55-0.89 |
| Gervasoni 2015 ^13^ | NA; AUC=0.72 (0.56-0.87) (fall); AUC=0.84 (0.72,0.97) (recurrent fall) | No information |
| Gu 2020 ^14^ | p-value=0.6, calibration curve was above idea calibration line; AUC= 0.94 (0.89,0.99) (XGBoost). p-value=0.96, calibration curve was closed to idea calibration line; AUC=0.89 (0.82,0.95) (logistics regression) | Cut points were model driven at 0.237 for XGBoost and at 0.155 for Logistics regression |
| Kelly 2019 ^15^ (external model validation study) | NA; C-statistic = 0.68 (0.55,0.81) | No information |
| Kerr 2010 ^16^ | NA; AUC=0.74 | No information |
| Lindholm 2016 ^17^ | Hosmer-Lemeshow test p-value=0.91; AUC=0.82 (0.75,0.89) (Not true external validation). p-value=0.90; only has AUC of the univariable analysis instead of the final model | No information |
| Liu 2017 ^18^ | NA; AUC=0.83 (0.79-0.88) (global cognitive impairment). NA; AUC=0.87 (0.82-0.92) in test set (dementia) | Predefine cut off = 0.196 (global cognitive impairment/dementia) |
| Lo 2019 ^19^ | NA; AUC = 0.79-0.94 (fall), 0.75-0.95 (freezing of gait), 0.79-0.91 (postural instability); 0.81-0.97 (cognitive impairment); 0.78-0.93 (difficulty doing hobbies); 0.83-0.99 (functional impairment) | No information |
| Macleod 2018 (dev) ^20^ | Calibration plots showed good calibration. Mortality: AUC = 0.75 (0.70-0.80), AUC = 0.73 (0.68-0.78) (excluding Charlson score). Dependency: AUC =0.77 (0.72-0.82). Dependency or death: AUC=0.74 (0.69-0.79) | No information |
| Macleod 2018 (val) ^20^ | Calibration plot. Mortality: AUC = 0.76 (0.68-0.85), AUC = 0.78 (0.70-0.86) (excluding Charlson score); Dependency: AUC =0.68 (0.61-0.75); Dependency or death: AUC = 0.68 (0.62-0.75) | No information |
| Mak 2014 ^21^ | NA; NA | Overall accuracy 85.9% |
| Paul 2013 ^22^ | Hosmer-Lemeshow test p-value=0.33; AUC= 0.80 (0.73,0.86), zero-corrected AUC=0.78 | No information |
| Phongpreecha 2020 ^23^ | NA; C-statistics reported by boxplot | No information |
| Pouwels 2013 ^24^ | NA; C-statistic=0.69 (osteoporotic), C-statistic=0.73 (hip fracture) | No information |
| Redensek 2019 ^25^ | NA; AUC=0.7 (motor fluctuations [clinical model]), AUC=0.68 (motor fluctuations [clinical-pharmacogenetic model]), AUC= 0.71 (dyskinesia [clinical model]), AUC=0.68 (dyskinesia [clinical-pharmacogenetic model]) | Model driven threshold=-0.26 (motor fluctuations [clinical model]), threshold=-0.33 (motor fluctuations [clinical-pharmacogenetic model]), threshold=-1.51 (dyskinesia [clinical model]), threshold=-1.33 (dyskinesia [clinical-pharmacogenetic model]) |
| Schapira 2012 ^26^ | NA; C-statistic=0.70 | No information |
| Schrag 2017 ^27^ | Hosmer-Lemeshow test p-value=0.62; AUC=0.80 (bootstrap/cross-validation), AUC=0.76-0.82 (split data) | No information |
| Velseboer 2016 ^28^ | Calibration slope:0.91, calibration curves were close to idea calibration line; AUC= 0.75 (Internal validation). Calibration slope=1.13; AUC =0.85 (0.77,0.93) (External validation) | No information |
| Wang 2017 ^29^ | NA; AUC=0.75-0.79 by different time horizons | No information |
| Wang 2017 ^30^ | NA; ROC-AUC = 0.99 | No information |
| Ye 2017 ^31^ | NA; IAUC = 0.79 (0.73,0.85) | unclear whether a prior |

Abbreviation: NA=not applicable.

Supplementary table 8. Inclusion and exclusion criteria

| **First author/Year** | **Inclusion criteria** | **Exclusion criteria** |
| --- | --- | --- |
| Almeida 2016 ^7^ | Walk without assistance of another person, with or without an assistive device. | People with PD in H&Y stage V, neurological conditions other than PD, cognitive impairment (MMSE cut off scores based on the level of education of each participant) and co-morbidities that would affect balance. |
| Ashburn 2001 ^8^ | Walk independently and scored above eight on the MEAMS. | Other neurological or vestibular conditions. |
| Custodio 2016 ^9^ | Spanish speakers > 60 years old, ≥ 6 years education, able to walk independently and on stable doses of DRT. | Co-morbidites impairing balance or gait. Several further exclusions relating to other causes of parkinsonism, causes of cognitive impairment, presence of cerebrovascular disease, presence of depression, and use of certain drugs. |
| Duncan 2015 ^10^ | Age > 40 years old, between H&Y Stages I-IV, and scored ≥ 24 on the MMSE. | Atypical parkinsonism or had previous surgical intervention specifically for PD. |
| Ehgoetz Martens 2018 ^11^ | Available longitudinal data from at least 2 points approximately 6-24 months apart. | Continuing freezers. |
| Exarchos 2012 ^12^ | Not stated | Not stated |
| Gervasoni 2015 ^13^ | H&Y scale 2-4, at least one fall in the year before study, being able to stand in upright position for 30″ without assistance, being able to walk at least for 10 m with or without an assistive device, and MMSE > 24. | Not stated |
| Gu 2020 ^14^ | Age > 30 years old and diagnosed within two years, with asymmetric resting tremor or asymmetric bradykinesia or two of bradykinesia, resting tremor and rigidity. Not treated with any PD medications within 60 days of the baseline visit and not be expected to require PD medications within at least 6 months from baseline. | Lack of sufficient data for assessing the outcome measure and covariates of this sub-study at baseline assessment and at two-year and not having a DAT deficit on imaging. Patients with a GDS-15 score ≥ 5 at baseline. |
| Kelly 2019 ^15^ | Levodopa-naive at the start of the study and subsequently started on levodopa, no treatment changes in the 4 weeks preceding levodopa initiation, no recent amantadine, and naive to COM inhibitors before levodopa initiation | Participants who developed dementia within 1 year of diagnosis |
| Kerr 2010 ^16^ | Independently living and walk without the use of any aids. | Use of walking aids and medical reasons |
| Lindholm 2016 ^17^ | All people diagnosed with PD receiving care at a south Swedish university hospital during 2007–2013 | Age > 80 years old, inability to understand instructions, MMSE score < 24, inability to stand without support and severe comorbidity. |
| Liu 2017 ^18^ | Not stated | MMSE ≤ 25 at baseline, first study visit occurred ≥ 12 years from disease onset, and missing covariates. |
| Lo 2019 ^19^ | Diagnosed ≤ 3 years | Non-idiopathic parkinsonism, dementia preceding PD by one year suggestive of Dementia with Lewy Bodies, cognitive impairment precluding informed consent. Patients already experienced event(s). |
| Macleod 2018 ^20^ | All incident patients with idiopathic PD (both development and validation cohorts) | Drug-induced parkinsonism/other parkinsonian syndromes. Already dependent at baseline for outcome related dependency. |
| Mak 2014 ^21^ | Age > 40 years, medically stable, and able to walk 6m at least 3 times with or without an assistive device | Had a neurologic condition other than idiopathic PD; MMSE score < 24; postural hypotension, visual disturbance or vestibular dysfunction affecting balance; and/or a significant cardiovascular or musculoskeletal disorder limiting locomotion or balance. |
| Paul 2013 ^22^ | Age >40 years and able to walk independently with or without an aid | MMSE score < 24 or suffered from any unstable cardiovascular, orthopaedic, or neurological conditions. |
| Phongpreecha 2020 ^23^ | Attend cognitive exanimation in the baseline and at least one follow-up exanimation. | Atypical parkinsonism, with an unknown/other cognitive diagnosis or those who were diagnosed with PDD but later reverted to PD-NCI or PD-MCI. |
| Pouwels 2013 ^24^ | Age ≥ 40 years in CPRD data collection 1987-2011, at least two records of a prescription for anti-Parkinson medication after diagnosis or COMT inhibitors. | Record for a prescription of PD treatment ≥1 before PD diagnosis, had been treated for osteoporosis. |
| Redensek 2019 ^25^ | Available clinical data, at least 3 months of levodopa treatment duration, ongoing dopaminergic therapy | Not stated |
| Schapira 2012 ^26^ | Aged 30-70 years and a disease duration of < 5 years from time of diagnosis. Could be taking stable doses of a dopamine agonist or other antiparkinsonian medications (no change in previous 4 weeks) but could not have taken amantadine within the preceding 270 days. | Atypical or secondary parkinsonism, con-comitant use of neuroleptic agents, prior neurosurgery for PD, and medical or psychiatric conditions that could interfere with the conduct of the study. Prior exposure to L-dopa for >30 days or within 8 weeks prior to entry, and previous use of a COMT inhibitor. |
| Schrag 2017 ^27^ | Age >30 years old; have an asymmetric resting tremor or asymmetric bradykinesia, or two of the three signs of bradykinesia, resting tremor, and rigidity; recently diagnosed of PD; be in H&Y stage 1 or 2. | Clinically significant neurological disorders, a first degree relative with PD, MOCA score ≤ 26, medication intake that might interfere with DAT imaging or preclude CSF collection, and use of investigational drugs or devices within 60 days of the baseline visit. |
| Velseboer 2016 ^28^ | Consecutive patients with newly diagnosed PD recruited from the neurology outpatient clinics of six general hospitals in the Netherlands (dev). Patients with newly diagnosed parkinsonism and resident in Cambridgeshire at the time of diagnosis (val). | Patients who already met the criteria of an unfavourable outcome at baseline, missing with outcomes or test in the baseline. |
| Wang 2017 ^29^ | Not stated | Not stated |
| Wang 2017 ^30^ | PD <10 years duration | Major comorbidities |
| Ye 2017 ^31^ | Exhibited significant dopamine transporter loss compatible with PD on F-FP-CIT PET scans and a positive motor response to dopaminergic medication | A current or past history of neurological or psychiatric illnesses. Patients with severe hearing impairment, visual loss, aphasia, malignancy, severe cardiac disorders, respiratory illnesses, hepatic disorders, and renal disorders. Subjects who had structural lesions including brain tumours, traumatic brain injuries, and hydrocephalus identified by MRI. |

Abbreviation: COM= Catechol-O-methyl; COMT= Catechol-O-methyl transferase; CPRD=Clinical Practice Research Datalink; CSF=cerebrospinal fluid; DAT=dopamine active transporter; dev=development; DRT=Dopamine replacement therapy; GDS= Geriatric Depression Scale; H&Y= Hoehn and Yahr; MEAMS=Middlesex Elderly Assessment of Mental State; MMSE=Mini-Mental State Examination; MOCA=Montreal Cognitive Assessment; PD=Parkinson’s disease; PDD=Parkinson disease with dementia; PD-MCI= Parkinson disease with mild cognitive impairment; PD-NCI= Parkinson disease with no cognitive impairment; PET=Positron Emission Tomography; val=validation.

Supplementary table 9. Participants (domain 1) in PROBAST

| **First author/Year** | **Appropriate data sources** | **Appropriate inclusion and exclusions of participants** | **Risk of bias** | **Applicability** |
| --- | --- | --- | --- | --- |
| Almeida 2016 ^7^ |  |  |  |  |
| Ashburn 2001 ^8^ |  |  |  |  |
| Custodio 2016 ^9^ |  |  |  |  |
| Duncan 2015 ^10^ |  |  |  |  |
| Ehgoetz Martens 2018 ^11^ |  |  |  |  |
| Exarchos 2012 ^12^ |  |  |  |  |
| Gervasoni 2015 ^13^ |  |  |  |  |
| Gu 2020 (XGBoost) ^14^ |  |  |  |  |
| Gu 2020 (Logistic regression) ^14^ |  |  |  |  |
| Kelly 2019 ^15^ |  |  |  |  |
| Kerr 2010 ^16^ |  |  |  |  |
| Lindholm 2016 ^17^ |  |  |  |  |
| Liu 2017 (Model development) ^18^ |  |  |  |  |
| Liu 2017 (Model validation) ^18^ |  |  |  |  |
| Lo 2019 ^19^ |  |  |  |  |
| Macleod 2018 (Model development) ^20^ |  |  |  |  |
| Macleod 2018 (Model validation) ^20^ |  |  |  |  |
| Mak 2014 ^21^ |  |  |  |  |
| Paul 2013 ^22^ |  |  |  |  |
| Phongpreecha 2020 ^23^ |  |  |  |  |
| Pouwels 2013 ^24^ |  |  |  |  |
| Redensek 2019 ^25^ |  |  |  |  |
| Schapira 2012 ^26^ |  |  |  |  |
| Schrag 2017 ^27^ |  |  |  |  |
| Velseboer 2016 (Model development) ^28^ |  |  |  |  |
| Velseboer 2016 (Model validation) ^28^ |  |  |  |  |
| Wang 2017 ^29^ |  |  |  |  |
| Wang 2017 ^30^ |  |  |  |  |
| Ye 2017 ^31^ |  |  |  |  |

Guide to colour shading in the following PROBAST tables.

| Yes | Probably yes | No | Probably no | No information |
| --- | --- | --- | --- | --- |
|  |  |  |  |  |
| Low/Low concern | High/High concern | Unclear/Unclear concern |  |  |
|  |  |  |  |  |

Supplementary table 10. Information on study participants

| **First author/Year** | **Recruitment method** | **Recruitment dates** | **Recruitment strategy** | **Diagnostic criteria** |
| --- | --- | --- | --- | --- |
| Almeida 2016 ^7^ | Not stated | Apr 2010 - Jun 2013 | Hospital clinic-based | UKPDBB |
| Ashburn 2001 ^8^ | Prevalence cohort | No information | Community/population-based | Not stated |
| Custodio 2016 ^9^ | Consecutive patients | Apr 2012 - Apr 2015 | Hospital clinic-based | UKPDBB |
| Duncan 2015 ^10^ | Other inception cohort | No information | Not stated | Not stated |
| Ehgoetz Martens 2018 ^11^ | Not stated | 2008 - 2016 | Hospital clinic-based | UKPDBB |
| Exarchos 2012 ^12^ | Not stated | No information | Hospital clinic-based | Not stated |
| Gervasoni 2015 ^13^ | Consecutive patients | No information | Not stated | Not stated |
| Gu 2020 ^14^ | Other inception cohort | Jun 2010 - Apr 2013 | Hospital clinic-based | Other |
| Kelly 2019 ^15^ | Other inception cohort | Sep 2010 - Sep 2014 | Hospital clinic-based | UKPDBB |
| Kerr 2010 ^16^ | Not stated | Mar 2002 - Dec 2006 | Community/population-based | Not stated |
| Lindholm 2016 ^17^ | Prevalence cohort | 2007-2013 | Hospital clinic-based | Not stated |
| Liu 2017 ^18^ | Other | Nov 1986 - Jun 2016 | Other | Other |
| Lo 2019 ^19^ | Other inception cohort | Aug 2014 - Nov 2017 | Hospital clinic-based | UKPDBB |
| Macleod 2018 ^20^ | Incidence cohort | 18-month from Nov 2002 & 36-month from Apr 2006 (dev); 22-month from Nov 2004 (val) | Community/population-based | UKPDBB |
| Mak 2014 ^21^ | Not stated | No information | Other | UKPDBB |
| Paul 2013 ^22^ | Not stated | No information | Not stated | Not stated |
| Phongpreecha 2020 ^23^ | Not stated | No information | Not stated | UKPDBB |
| Pouwels 2013 ^24^ | Other inception cohort | 1987 - 2011 | Community/population-based | Other |
| Redensek 2019 ^25^ | Not stated | Oct 2016 - Apr 2018 | Not stated | UKPDBB |
| Schapira 2012 ^26^ | Not stated | No information | Not stated | UKPDBB |
| Schrag 2017 ^27^ | Other inception cohort | Jul 2010 - May 2013 | Hospital clinic-based | Other |
| Velseboer 2016 ^28^ | Inception cohort (dev), incidence cohort (val) | Jul 2002 - Apr 2005 (dev); Dec 2000 - Dec 2002 (val) | Hospital clinic-based (dev), Community/population -based (val) | Other |
| Wang 2017 ^29^ | Not stated | No information | Hospital clinic-based | Not stated |
| Wang 2017 ^30^ | Not stated | No information | Hospital clinic-based | UKPDBB |
| Ye 2017 ^31^ | Not stated | Mar 2007 - Dec 2013 | Hospital clinic-based | UKPDBB |

Abbreviation: UKPDBB=UK Parkinson's disease brain bank criteria; dev=development; val=validation.

Supplementary table 11. Predictors (domain 2) in PROBAST

| **First author/Year** | **Predictor definition** | **Predictors assessments made without knowledge of outcome data** | **All predictors are available at the time the model is intended to be used** | **Risk of bias** | **Applicability** |
| --- | --- | --- | --- | --- | --- |
| Almeida 2016 ^7^ |  |  |  |  |  |
| Ashburn 2001 ^8^ |  |  |  |  |  |
| Custodio 2016 ^9^ |  |  |  |  |  |
| Duncan 2015 ^10^ |  |  |  |  |  |
| Ehgoetz Martens 2018 ^11^ |  |  |  |  |  |
| Exarchos 2012 ^12^ |  |  |  |  |  |
| Gervasoni 2015 ^13^ |  |  |  |  |  |
| Gu 2020 (XGBoost) ^14^ |  |  |  |  |  |
| Gu 2020 (Logistic regression) ^14^ |  |  |  |  |  |
| Kelly 2019 ^15^ |  |  |  |  |  |
| Kerr 2010 ^16^ |  |  |  |  |  |
| Lindholm 2016 ^17^ |  |  |  |  |  |
| Liu 2017 (Model development) ^18^ |  |  |  |  |  |
| Liu 2017 (Model validation) ^18^ |  |  |  |  |  |
| Lo 2019 ^19^ |  |  |  |  |  |
| Macleod 2018 (Model development) ^20^ |  |  |  |  |  |
| Macleod 2018 (Model validation) ^20^ |  |  |  |  |  |
| Mak 2014 ^21^ |  |  |  |  |  |
| Paul 2013 ^22^ |  |  |  |  |  |
| Phongpreecha 2020 ^23^ |  |  |  |  |  |
| Pouwels 2013 ^24^ |  |  |  |  |  |
| Redensek 2019 ^25^ |  |  |  |  |  |
| Schapira 2012 ^26^ |  |  |  |  |  |
| Schrag 2017 ^27^ |  |  |  |  |  |
| Velseboer 2016 (Model development) ^28^ |  |  |  |  |  |
| Velseboer 2016 (Model validation) ^28^ |  |  |  |  |  |
| Wang 2017 ^29^ |  |  |  |  |  |
| Wang 2017 ^30^ |  |  |  |  |  |
| Ye 2017 ^31^ |  |  |  |  |  |

Footnote: Whether all predictors are available at the time the model is intended to be used is only relevant to applicability.

Guide to colour shading in the following PROBAST tables.

| Yes | Probably yes | No | Probably no | No information |
| --- | --- | --- | --- | --- |
|  |  |  |  |  |
| Low/Low concern | High/High concern | Unclear/Unclear concern |  |  |
|  |  |  |  |  |

Supplementary table 12. Outcome(s) in studies

| **First author/Year** | **Outcomes** | **Definition of outcomes** |
| --- | --- | --- |
| Almeida 2016 ^7^ | Falls | A fall was defined as “an event which results in a person coming to rest unintentionally on the ground or other lower level, not as the result of a major intrinsic event or overwhelming hazard”. |
| Ashburn 2001 ^8^ | Falls | A fall was defined as “an event which results in a person coming to rest unintentionally on the ground or other lower level, not as the result of a major intrinsic event or overwhelming hazard”. |
| Custodio 2016 ^9^ | Falls | Unintentionally coming to rest on the ground or other lower surface without overwhelming external force or a major internal event. |
| Duncan 2015 ^10^ | Falls | Unintentionally coming to rest on the ground or other lower surface without overwhelming external force or a major internal event. |
| Ehgoetz Martens 2018 ^11^ | Freezing of gait | Continuing freezers were defined as freezing of Gait-Questionnaire (FOG-Q3) >1 at baseline and follow-up; transitional freezers were defined FOG-Q3 = 0 at baseline and >1 at follow-up. |
| Exarchos 2012 ^12^ | Tremor, rigidity, body bradykinesia, postural instability, falls, freezing of gait, autonomic symptoms, hypophonia, hypomimia, orthostatic hypotension, REM sleep behavioural disorder, dyskinesias, motor fluctuations, sudden motor fluctuations and dementia | Not stated |
| Gervasoni 2015 ^13^ | Falls and recurrent falls | A fall was defined as “an episode of unintentionally coming to rest on the ground or lower surface that was not the result of dizziness, fainting, sustaining a violent blow, loss of consciousness, or other overwhelming external factor”. Subjects with at least one fall in the 6 months after the beginning of the study were categorized as fallers; recurrent fallers were those with two or more falls in the same period. |
| Gu 2020 ^14^ | Depression | Score ≥5 on the 15-item Geriatric Depression Scale score. |
| Kelly 2019 ^15^ | Dyskinesia | The presence and intensity of motor complications was determined using the Movement Disorder Society– Sponsored Revision of the Unified Parkinson’s Disease Rating Scale (MDS-UPDRS) IV questions 1 (dyskinesia). Dyskinesia was also recorded if witnessed by the interviewer. |
| Kerr 2010 ^16^ | Falls | A fall was defined as unintentionally coming to the ground or some lower level not as a result of a major intrinsic event (e.g., stroke) or overwhelming hazard. |
| Lindholm 2016 ^17^ | Falls | Falls were defined as ‘‘an unexpected event in which the participants come to rest on the ground, floor, or lower level”. |
| Liu 2017 ^18^ | Global cognitive impairment and dementia within 10 years from onset | MMSE scores with the cutoff of 25 or less was defined as an indicator of substantial global cognitive impairment. Dementia was defined in different ways in different cohorts (can be found in paper’s Supplementary appendix table e1) |
| Lo 2019 ^19^ | Falls, freezing of gait, postural instability, cognitive impairment, difficulty doing hobbies, functional dependency | (1) falls (>1 self-reported fall in the preceding 6 months), (2) freezing (a freezing frequency of at least “about once a month” on the FOG questionnaire), (3) Postural instability (Hoehn and Yahr stage ≥ 3), (4) Cognitive impairment (a Montreal Cognitive Assessment score < 26), (5) Difficulty doing hobbies (Movement Disorders Society Unified Parkinson’s Disease Rating Scale part II item 2.8 score ≥ 3 indicating major difficulty or an inability to do activities of enjoyment) and (6) the self-reported need for future help at home. |
| Macleod 2018 ^20^ | Mortality, functional dependency, death or dependency | Data on mortality were derived from notifications by relatives/general practitioners plus surveillance by the UK national death registers in the PINE study. Functional dependency was measured by the Schwab & England scale at follow-up visits and defined using a cut-off of < 80% (80% = completely independent in most chores; 70% = not completely independent). The word chores was consistently interpreted as basic activities of daily living (walking, personal hygiene, dressing, toileting, feeding) by both study teams. |
| Mak 2014 ^21^ | Recurrent falls | Those who experienced >1 fall within the 12-month follow up period were classified as recurrent fallers. |
| Paul 2013 ^22^ | Falls | A fall was defined as unintentionally coming to rest on the ground or other lower surface without overwhelming external force or a major internal event. |
| Phongpreecha 2020 ^23^ | No cognitive impairment, mild cognitive impairment, dementia | Mild cognitive impairment was defined by Movement Disorders Society PD-MCI Level II criteria. |
| Pouwels 2013 ^24^ | Osteoporotic and hip fracture risks | A clinical osteoporotic fracture was defined as a clinically symptomatic fracture of the radius/ulna, humerus, rib, femur/hip, pelvis, or vertebrae. The fracture types were classified according to the International Classification of Diseases, Tenth Revision (ICD-10) categories. |
| Redensek 2019 ^25^ | Motor fluctuations and dyskinesia | Motor fluctuations present as oscillations between good motor symptom control and reduced motor symptom control, while dyskinesia manifest as involuntary choreatic or dystonic movements. |
| Schapira 2012 ^26^ | Dyskinesias | The primary endpoint was the time to onset of dyskinesia. Dyskinesia was determined by the blinded rater based on either direct observation or by patient response to specific questions. |
| Schrag 2017 ^27^ | Cognitive impairment | Mild cognitive impairment was defined as scores on two or more of the (Hopkins Verbal Learning Test-Revised) HVLT total recall, HVLT recognition discrimination, Benton Judgment of Line Orientation, Letter-Number Sequencing, semantic (animal) fluency test, or Symbol-Digit Modalities Test of more than 1.5 standard deviations below normal, and no functional impairment due to cognition impairment. A diagnosis of dementia also required evidence of functional impairment attributable to cognitive impairment sufficient to interfere with activities of daily life. |
| Velseboer 2016 ^28^ | Composite poor outcome (postural instability, dementia, or death) | The presence of postural instability was assessed on the basis of a modified Hoehn & Yahr scale score of 3 or higher. Patients were classified as having dementia using level 1 criteria from the Movement Disorder Society Task Force, operationalized using the Mini-Mental State Examination in addition to the clock drawing test in the CARPA study and a phonemic fluency test in the CamPaIGN study. |
| Wang 2017 ^29^ | Need for levodopa treatment | Patients reached a pre-defined level of functional disability, which is considered to be a terminal event because these patients would then initiate symptomatic treatment of levodopa. |
| Wang 2017 ^30^ | Imbalance | Imbalance is defined as Hoehn & Yahr scale >1 or item 2.12 from the Movement Disorder Society Revision of the Unified Parkinson Disease Rating Scale part 2 (issues with walking and balance) was > = 3. |
| Ye 2017 ^31^ | Dementia | Movement Disorder Society Task Force Parkinson’s disease dementia criteria. |

| Supplementary table 13. Outcomes (domain 3) in PROBAST   \| **First author/Year** \| **Appropriate outcome** \| **A prespecified outcome definition** \| **Predictors excluded from the outcome definition** \| **Outcome defined and determined in a similar way for all participant** \| **The outcome determined without knowledge of predictor information** \| **Appropriate time interval between predictor assessment and outcome** \| **Risk of bias** \| **Applicability** \| \| --- \| --- \| --- \| --- \| --- \| --- \| --- \| --- \| --- \| \| Almeida 2016 ^7^ \|  \|  \|  \|  \|  \|  \|  \|  \| \| Ashburn 2001 ^8^ \|  \|  \|  \|  \|  \|  \|  \|  \| \| Custodio 2016 ^9^ \|  \|  \|  \|  \|  \|  \|  \|  \| \| Duncan 2015 ^10^ \|  \|  \|  \|  \|  \|  \|  \|  \| \| Ehgoetz Martens 2018 ^11^ \|  \|  \|  \|  \|  \|  \|  \|  \| \| Exarchos 2012 ^12^ \|  \|  \|  \|  \|  \|  \|  \|  \| \| Gervasoni 2015 ^13^ \|  \|  \|  \|  \|  \|  \|  \|  \| \| Gu 2020 (XGBoost) ^14^ \|  \|  \|  \|  \|  \|  \|  \|  \| \| Gu 2020 (Logistic regression) ^14^ \|  \|  \|  \|  \|  \|  \|  \|  \| \| Kelly 2019 ^15^ \|  \|  \|  \|  \|  \|  \|  \|  \| \| Kerr 2010 ^16^ \|  \|  \|  \|  \|  \|  \|  \|  \| \| Lindholm 2016 ^17^ \|  \|  \|  \|  \|  \|  \|  \|  \| \| Liu 2017 (Model development) ^18^ \|  \|  \|  \|  \|  \|  \|  \|  \| \| Liu 2017 (Model validation) ^18^ \|  \|  \|  \|  \|  \|  \|  \|  \| \| Lo 2019 ^19^ \|  \|  \|  \|  \|  \|  \|  \|  \| \| Macleod 2018 (Model development) ^20^ \|  \|  \|  \|  \|  \|  \|  \|  \| \| Macleod 2018 (Model validation) ^20^ \|  \|  \|  \|  \|  \|  \|  \|  \| \| Mak 2014 ^21^ \|  \|  \|  \|  \|  \|  \|  \|  \| \| Paul 2013 ^22^ \|  \|  \|  \|  \|  \|  \|  \|  \| \| Phongpreecha 2020 ^23^ \|  \|  \|  \|  \|  \|  \|  \|  \| \| Pouwels 2013 ^24^ \|  \|  \|  \|  \|  \|  \|  \|  \| \| Redensek 2019 ^25^ \|  \|  \|  \|  \|  \|  \|  \|  \| \| Schapira 2012 ^26^ \|  \|  \|  \|  \|  \|  \|  \|  \| \| Schrag 2017 ^27^ \|  \|  \|  \|  \|  \|  \|  \|  \| \| Velseboer 2016 (Model development) ^28^ \|  \|  \|  \|  \|  \|  \|  \|  \| \| Velseboer 2016 (Model validation) ^28^ \|  \|  \|  \|  \|  \|  \|  \|  \| \| Wang 2017 ^29^ \|  \|  \|  \|  \|  \|  \|  \|  \| \| Wang 2017 ^30^ \|  \|  \|  \|  \|  \|  \|  \|  \| \| Ye 2017 ^31^ \|  \|  \|  \|  \|  \|  \|  \|  \| |
| --- | --- | --- | --- | --- | --- | --- | --- | --- | --- | --- | --- | --- | --- | --- | --- | --- | --- | --- | --- | --- | --- | --- | --- | --- | --- | --- | --- | --- | --- | --- | --- | --- | --- | --- | --- | --- | --- | --- | --- | --- | --- | --- | --- | --- | --- | --- | --- | --- | --- | --- | --- | --- | --- | --- | --- | --- | --- | --- | --- | --- | --- | --- | --- | --- | --- | --- | --- | --- | --- | --- | --- | --- | --- | --- | --- | --- | --- | --- | --- | --- | --- | --- | --- | --- | --- | --- | --- | --- | --- | --- | --- | --- | --- | --- | --- | --- | --- | --- | --- | --- | --- | --- | --- | --- | --- | --- | --- | --- | --- | --- | --- | --- | --- | --- | --- | --- | --- | --- | --- | --- | --- | --- | --- | --- | --- | --- | --- | --- | --- | --- | --- | --- | --- | --- | --- | --- | --- | --- | --- | --- | --- | --- | --- | --- | --- | --- | --- | --- | --- | --- | --- | --- | --- | --- | --- | --- | --- | --- | --- | --- | --- | --- | --- | --- | --- | --- | --- | --- | --- | --- | --- | --- | --- | --- | --- | --- | --- | --- | --- | --- | --- | --- | --- | --- | --- | --- | --- | --- | --- | --- | --- | --- | --- | --- | --- | --- | --- | --- | --- | --- | --- | --- | --- | --- | --- | --- | --- | --- | --- | --- | --- | --- | --- | --- | --- | --- | --- | --- | --- | --- | --- | --- | --- | --- | --- | --- | --- | --- | --- | --- | --- | --- | --- | --- | --- | --- | --- | --- | --- | --- | --- | --- | --- | --- | --- | --- | --- | --- | --- | --- | --- | --- | --- | --- | --- | --- | --- | --- | --- | --- | --- | --- | --- | --- | --- | --- | --- | --- | --- | --- |

Guide to colour shading in the following PROBAST tables.

| Yes | Probably yes | No | Probably no | No information |
| --- | --- | --- | --- | --- |
|  |  |  |  |  |
| Low/Low concern | High/High concern | Unclear/Unclear concern |  |  |
|  |  |  |  |  |

Supplementary table 14. Analysis (domain 4) in PROBAST

| \| **First author/Year** \| **Reasonable number of participants with the outcome** \| **Appropriately handle continuous predictors** \| **All enrolled participants included in the analysis** \| **Appropriately handle the missing data** \| **Avoid selecting the predictors based on univariable analysis** \| **Appropriately account for censoring in the data** \| **Model performance measures evaluated appropriately** \| **Account for models’ overfitting** \| **Present full equation of the final model** \| **Risk of bias** \| \| --- \| --- \| --- \| --- \| --- \| --- \| --- \| --- \| --- \| --- \| --- \| \| Almeida 2016 ^7^ \|  \|  \|  \|  \|  \|  \|  \|  \|  \|  \| \| Ashburn 2001 ^8^ \|  \|  \|  \|  \|  \|  \|  \|  \|  \|  \| \| Custodio 2016 ^9^ \|  \|  \|  \|  \|  \|  \|  \|  \|  \|  \| \| Duncan 2015 ^10^ \|  \|  \|  \|  \|  \|  \|  \|  \|  \|  \| \| Ehgoetz Martens 2018 ^11^ \|  \|  \|  \|  \|  \|  \|  \|  \|  \|  \| \| Exarchos 2012 ^12^ \|  \|  \|  \|  \|  \|  \|  \|  \|  \|  \| \| Gervasoni 2015 ^13^ \|  \|  \|  \|  \|  \|  \|  \|  \|  \|  \| \| Gu 2020 (XGBoost) ^14^ \|  \|  \|  \|  \|  \|  \|  \|  \|  \|  \| \| Gu 2020 (Logistic regression) ^14^ \|  \|  \|  \|  \|  \|  \|  \|  \|  \|  \| \| Kelly 2019 ^15^ \|  \|  \|  \|  \| Not applicable \|  \|  \| Not applicable \| Not applicable \|  \| \| Kerr 2010 ^16^ \|  \|  \|  \|  \|  \|  \|  \|  \|  \|  \| \| Lindholm 2016 ^17^ \|  \|  \|  \|  \|  \|  \|  \|  \|  \|  \| \| Liu 2017 (Model development) ^18^ \|  \|  \|  \|  \|  \|  \|  \|  \|  \|  \| \| Liu 2017 (Model validation) ^18^ \|  \|  \|  \|  \| Not applicable \|  \|  \| Not applicable \| Not applicable \|  \| \| Lo 2019 ^19^ \|  \|  \|  \|  \|  \|  \|  \|  \|  \|  \| \| Macleod 2018 (Model development) ^20^ \|  \|  \|  \|  \|  \|  \|  \|  \|  \|  \| \| Macleod 2018 (Model validation) ^20^ \|  \|  \|  \|  \| Not applicable \|  \|  \| Not applicable \| Not applicable \|  \| \| Mak 2014 ^21^ \|  \|  \|  \|  \|  \|  \|  \|  \|  \|  \| \| Paul 2013 ^22^ \|  \|  \|  \|  \|  \|  \|  \|  \|  \|  \| \| Phongpreecha 2020 ^23^ \|  \|  \|  \|  \|  \|  \|  \|  \|  \|  \| \| Pouwels 2013 ^24^ \|  \|  \|  \|  \|  \|  \|  \|  \|  \|  \| \| Redensek 2019 ^25^ \|  \|  \|  \|  \|  \|  \|  \|  \|  \|  \| \| Schapira 2012 ^26^ \|  \|  \|  \|  \|  \|  \|  \|  \|  \|  \| \| Schrag 2017 ^27^ \|  \|  \|  \|  \|  \|  \|  \|  \|  \|  \| \| Velseboer 2016 (Model development) ^28^ \|  \|  \|  \|  \|  \|  \|  \|  \|  \|  \| \| Velseboer 2016 (Model validation) ^28^ \|  \|  \|  \|  \| Not applicable \|  \|  \| Not applicable \| Not applicable \|  \| \| Wang 2017 ^29^ \|  \|  \|  \|  \|  \|  \|  \|  \|  \|  \| \| Wang 2017 ^30^ \|  \|  \|  \|  \|  \|  \|  \|  \|  \|  \| \| Ye 2017 ^31^ \|  \|  \|  \|  \|  \|  \|  \|  \|  \|  \|   Footnote: R. P. Duncan and B. Lindholm viewed as model development studies instead of external validation studies in PROBAST to assess the risk of bias.  Guide to colour shading in the following PROBAST tables.   \| Yes \| Probably yes \| No \| Probably no \| No information \| \| --- \| --- \| --- \| --- \| --- \| \|  \|  \|  \|  \|  \| \| Low/Low concern \| High/High concern \| Unclear/Unclear concern \|  \|  \| \|  \|  \|  \|  \|  \| |
| --- | --- | --- | --- | --- | --- | --- | --- | --- | --- | --- | --- | --- | --- | --- | --- | --- | --- | --- | --- | --- | --- | --- | --- | --- | --- | --- | --- | --- | --- | --- | --- | --- | --- | --- | --- | --- | --- | --- | --- | --- | --- | --- | --- | --- | --- | --- | --- | --- | --- | --- | --- | --- | --- | --- | --- | --- | --- | --- | --- | --- | --- | --- | --- | --- | --- | --- | --- | --- | --- | --- | --- | --- | --- | --- | --- | --- | --- | --- | --- | --- | --- | --- | --- | --- | --- | --- | --- | --- | --- | --- | --- | --- | --- | --- | --- | --- | --- | --- | --- | --- | --- | --- | --- | --- | --- | --- | --- | --- | --- | --- | --- | --- | --- | --- | --- | --- | --- | --- | --- | --- | --- | --- | --- | --- | --- | --- | --- | --- | --- | --- | --- | --- | --- | --- | --- | --- | --- | --- | --- | --- | --- | --- | --- | --- | --- | --- | --- | --- | --- | --- | --- | --- | --- | --- | --- | --- | --- | --- | --- | --- | --- | --- | --- | --- | --- | --- | --- | --- | --- | --- | --- | --- | --- | --- | --- | --- | --- | --- | --- | --- | --- | --- | --- | --- | --- | --- | --- | --- | --- | --- | --- | --- | --- | --- | --- | --- | --- | --- | --- | --- | --- | --- | --- | --- | --- | --- | --- | --- | --- | --- | --- | --- | --- | --- | --- | --- | --- | --- | --- | --- | --- | --- | --- | --- | --- | --- | --- | --- | --- | --- | --- | --- | --- | --- | --- | --- | --- | --- | --- | --- | --- | --- | --- | --- | --- | --- | --- | --- | --- | --- | --- | --- | --- | --- | --- | --- | --- | --- | --- | --- | --- | --- | --- | --- | --- | --- | --- | --- | --- | --- | --- | --- | --- | --- | --- | --- | --- | --- | --- | --- | --- | --- | --- | --- | --- | --- | --- | --- | --- | --- | --- | --- | --- | --- | --- | --- | --- | --- | --- | --- | --- | --- | --- | --- | --- | --- | --- | --- | --- | --- | --- | --- | --- | --- | --- | --- | --- | --- | --- | --- | --- | --- | --- | --- | --- | --- | --- | --- | --- | --- | --- | --- | --- | --- | --- | --- | --- | --- | --- | --- | --- | --- | --- | --- | --- | --- | --- | --- | --- | --- |
